# Adolescent girls and young women’s PrEP-user journey during an implementation science study in South Africa and Kenya

**DOI:** 10.1371/journal.pone.0258542

**Published:** 2021-10-14

**Authors:** Elzette Rousseau, Ariana W. K. Katz, Shannon O’Rourke, Linda-Gail Bekker, Sinead Delany-Moretlwe, Elizabeth Bukusi, Danielle Travill, Victor Omollo, Jennifer F. Morton, Gabrielle O’Malley, Jessica E. Haberer, Renee Heffron, Rachel Johnson, Connie Celum, Jared M. Baeten, Ariane van der Straten

**Affiliations:** 1 Desmond Tutu HIV Centre, University of Cape Town, Cape Town, South Africa; 2 RTI International, Women’s Global Health Imperative (WGHI), Berkeley, CA, United States of America; 3 Wits RHI, University of the Witwatersrand, Johannesburg, South Africa; 4 Kenya Medical Research Institute, Kisumu, Kenya; 5 International Clinical Research Center, University of Washington, Seattle, WA, United States of America; 6 Harvard Medical School, Harvard University, Boston, MA, United States of America; 7 Centre for Global Health, Massachusetts General Hospital, Boston, MA, United States of America; 8 Gilead Sciences, Inc., Seattle, WA, United States of America; 9 Centre for AIDS Prevention Studies, Department of Medicine, University of California, San Francisco, CA, United States of America; Washington University in Saint Louis, UNITED STATES

## Abstract

Successful scale-up of PrEP for HIV prevention in African adolescent girls and young women (AGYW) requires integration of PrEP into young women’s everyday lives. We conducted interviews and focus group discussions with 137 AGYW PrEP users aged 16–25 from South Africa and Kenya. Individual and relational enablers and disablers were explored at key moments during their PrEP-user journey from awareness, initiation and early use through persistence, including PrEP pauses, restarts, and discontinuation. PrEP uptake was facilitated when offered as part of an integrated sexual reproductive health service, but hampered by low awareness, stigma and misconceptions about PrEP in the community. Daily pill-taking was challenging for AGYW due to individual, relational and structural factors and PrEP interruptions (intended or unintended) were described as part of AGYW’s PrEP-user journey. Disclosure, social support, adolescent-friendly health counseling, and convenient access to PrEP were reported as key enablers for PrEP persistence.

## Introduction

Globally, 1000 new infections occur daily in adolescent girls and young women (AGYW, ages 15–24), and HIV is the leading cause of death for women (15–49 years) [1]. In sub-Saharan Africa (SSA), AGYW are disproportionately affected, accounting for 25% of all new infections. AGYW are 2.5 times more likely to acquire HIV through heterosexual sexual contact than their male peers [2]. Young people who are single or entering steady relationships are especially vulnerable to HIV acquisition [3]. Reducing HIV infection among AGYW is complicated by social factors that limit the ability of women to practice safer sex. These factors include a lack of communication between men and women regarding sexual health issues; gender inequality and violence in sexual relationships; difficulties with condom use negotiation; and overall low perception of HIV vulnerability in AGYW [4–7].

AGYW have welcomed oral pre-exposure prophylaxis (PrEP) as an HIV prevention method that will reduce HIV-related anxiety and increase their autonomy over their sexual health, independent of sexual partners’ knowledge or approval [8–10]. While daily dosing of oral PrEP (emtricitabine/tenofovir disoproxil fumarate) reduces the risk of acquiring HIV by more than 95% when used consistently [11,12], clinical trials in young African women have highlighted many barriers to use [13–15]. PrEP demonstration studies suggest that HIV-related stigma is pervasive, and confusing PrEP with antiretrovirals for treatment (ART) is common, creating a large deterrent to PrEP use [15–18]. Other barriers to PrEP uptake among AGYW include poor perception of HIV vulnerability, low awareness about PrEP, and lack of social support for PrEP use. Lower PrEP adherence has been noted among AGYW in relationships with intimate partner violence (IPV), in committed relationships where a sense of trust has been established, and situations where AGYW were hiding pill taking from their significant others [9,19–22]. Successful scale-up of PrEP in SSA will necessitate AGYW’s ability to integrate PrEP into their everyday lives. This includes oral PrEP use among AGYW becoming more normalized along with AGYW accurately assessing their vulnerability for HIV acquisition and aligning their PrEP use to periods of HIV risk in what is called prevention-effective adherence [23].

Previous research on female-initiated HIV prevention technologies indicated that PrEP use integration will entail a focus on accessibility and practicalities of use, as well as the personal and relationship interests that influence uptake and continued PrEP use [24,25]. During the POWER PrEP implementation science project in Kenya and South Africa, we conducted qualitative research to explore AGYW’s PrEP-user journey [26] from awareness and initiation of PrEP, to early use and persistence, including PrEP pauses, restarts, and discontinuation. This manuscript aims to highlight individual and relational enablers and disablers of PrEP engagement at each of these key moments along the PrEP user-journey.

## Materials and methods

### Research setting and study participants

The POWER (Prevention Options for Women Evaluation Research) implementation science study aimed to develop scalable and context specific PrEP delivery strategies for AGYW in Africa [27]. Between 2017–2020, 2550 AGYW enrolled on the POWER study at two family planning clinics in Kisumu, Kenya; an adolescent friendly clinic in Johannesburg; or a community mobile health clinic in Cape Town, South Africa. Inclusion criteria were being a cisgender woman, 16–25 years old (18–25 in Johannesburg), HIV uninfected, and sexually active at least once within the previous 3 months. Young women were given the option to start PrEP on the same day or delay PrEP initiation (PrEP initiation was not an enrollment requirement), with decision-making processes regarding PrEP uptake and persistence tracked for up to 36 months.

A subset of AGYW were purposively selected from this cohort for 104 in-depth interviews (IDIs) and six focus group discussions (FGDs; n = 33). Qualitative participants were recruited based on their decision to initiate PrEP and their persistence with PrEP refills throughout the study, as established through pharmacy records. Quota sampling was employed to ensure eligible participants were chosen for the IDI’s according to their unique PrEP-user journey experience and not necessarily to be representative of the overall POWER study cohort (Table 1). Using convenience sampling, participants who had at least one PrEP refill were recruited for FGD’s during clinic visits toward the end of their study participation.

**Table 1:** Participant categories.

Participant Category	Definition	Number qualitatively interviewed^a^ N = 137	Number in entire POWER cohort^b^ N = 2550 (%)
**Early Acceptors**	Participants who initiated PrEP at enrollment	24	2359 (93%)
**Initial Decliners**	Participants who declined PrEP at enrollment (but may or may not have started later)	23	183 (7%)
**Persistors**	Participants who initiated PrEP at enrollment and continued PrEP use over 6-month period with no gaps in pill coverage based on pill dispensing records	16	52 (3%)
**Non-Persistors**	Participants who initiated PrEP at enrollment and had a recently scheduled month 3 or month 6 visit and pharmacy records indicate late, missed or declined PrEP pill pick-up	23	2020 (92%)
**Restarters**	Participants who initiated PrEP and pharmacy records show a break in PrEP use for more than 30 days before a PrEP pill pick-up at a later clinic visit	5	384 (19%)
**Special Cases**	Participants whose unique circumstances or perspectives stood out and whose experiences could inform PrEP delivery, including participants who sero-converted to HIV	13	n/a
**FGD participant**	Participants who have had at least one PrEP refill were recruited for FGD’s during clinic visits toward the end of their study participation	33	n/a

Participants selected for IDI’s according to their unique PrEP-user journey experience based on PrEP pharmacy records.

^a^Participants could fall into more than one category, however participants here only indicated in categories originally recruited into.

^b^Celum, et al. (2021) [27].

The research was approved by the human research ethics committees of the University of Washington, the University of Cape Town, the Kenya Medical Research Institute, and the University of Witwatersrand. Prior to data collection, young women were informed about the focus and procedure of the research, and written informed consent was obtained in English or local languages (Xhosa, Zulu, Kiswahili, or Dhuluo). Parental consent was waived in Kisumu and Cape Town for participants younger than 18.

### Data collection

IDI (S1 Appendix) and FGD guides (S2 Appendix) were developed in English and translated into local languages (S3 Appendix). Interview guides included questions about the AGYW’s PrEP-user journey experiences, including their awareness and interest in PrEP; the enablers and disablers to PrEP uptake and use; the influence of family, peers, intimate partners and other community on PrEP use; and the key points of support in the PrEP-user journey. The FGD guide included the use of an adapted vignette, with the goal of getting young women to express their personal views of PrEP through the lens of a fictional persona (Lebo in South Africa; Anyango in Kenya). The guide included questions about the persona’s experiences, thoughts, and decisions to start, pause, restart and discontinue PrEP as well as probes to understand differences between the persona and the participants’ actual experiences.

All IDIs and FGDs were conducted face-to-face in the participants’ preferred language by experienced social science interviewers who were independent of the demonstration study’s clinical team. IDIs and FGDs lasted between 45–120 minutes and were audio recorded, then simultaneously translated and transcribed, and English transcripts were quality controlled by bilingual research assistants.

### Data analysis

An ‘end-user journey’ approach (based on the human-centered design framework: http://www.engagehcd.com/dpv-ring/) informed the codebook development [26]. The codebook was iteratively created to reflect the participants’ unique experiences at each moment of the PrEP-user journey: PrEP awareness, uptake, early use (month 0–3), persistence (beyond 3 months), pause and restart, and discontinuation. Additional codes were included to capture themes such as risk perception, disclosure, social support, relationship dynamics, and stigma/misconceptions to understand how these influenced decisions during the PrEP-user journey. Transcripts were coded in Dedoose (Version 6.1.18, Los Angeles, CA: Socio-Cultural Research Consultants, LLC) and analyzed thematically [28] to explore how AGYW fit PrEP into their lives and to understand AGYW’s needs at different key moments in their PrEP-user journey. Sections of independently coded transcripts were periodically compared by the four-person analysis team throughout the analysis process using the Dedoose Training Center with an average kappa of 0.76. Any disagreements were resolved through discussion until consensus was reached.

## Results

Participants in the POWER qualitative sub-study included 137 AGYW aged 16–25 (median age = 21) from Johannesburg (n = 46) and Cape Town (n = 43), South Africa and Kisumu (n = 48), Kenya who participated in IDI’s (n = 104) or FGD’s (n = 33). As illustrated in Table 2, the majority of young women were single with a partner (81%) and living with their parents or other family members (74%). Almost two-thirds of AGYW (63%) tested positive for a sexually transmitted infection (STI; either gonorrhea or chlamydia) at enrollment and 47% reported regular alcohol consumption. Seven young women (5%) were in known HIV serodiscordant relationships. Most young women receiving IDIs (59%; 61 of 104) had a PrEP interruption (i.e., following initiation, was not dispensed PrEP at an attended visit or was >14 days without pills due to a missed/late visit) during their use journey, with half (49%; 30 of 61) restarting PrEP within 30–60 days after pausing. Six young women interviewed seroconverted to HIV (either within the first month of PrEP use and could have been infected at PrEP initiation); or later in the PrEP user-journey after missing a PrEP refill or taking a PrEP pause.

**Table 2:** Demographic and behavioral characteristics of participants.

	*N = 137* (%)
**Age**	
16–19	44 (32.4)
20–25	92 (67.6)
**Relationship status**	
Single, with partner	110 (80.9)
Single, no partner	2 (1.5)
Married, husband has one wife	21 (15.4)
Married, husband has multiple wives	2 (1.5)
**Sexual partner’s HIV status**	
Sex partner is HIV+	7 (5.1)
HIV partner is not HIV+	48 (35.5)
Don’t know sex partner’s HIV status	81 (59.6)
**Living situation** ^**a**^	
Parents	66 (48.9)
Other family	34 (25.2)
Sex partner	23 (17.0)
Friends	1 (0.7)
Alone	15 (11.1)
Other	6 (4.4)
**Alcohol consumption**	
Yes	64 (47.1)
no	72 (52.9)
**STI at enrollment visit**	
Gonorrhea and/or chlamydia	71 (63.4)
None	41 (36.6)

^a^AGYW could mark more than one category (‘all that apply’).

**Fig 1:**
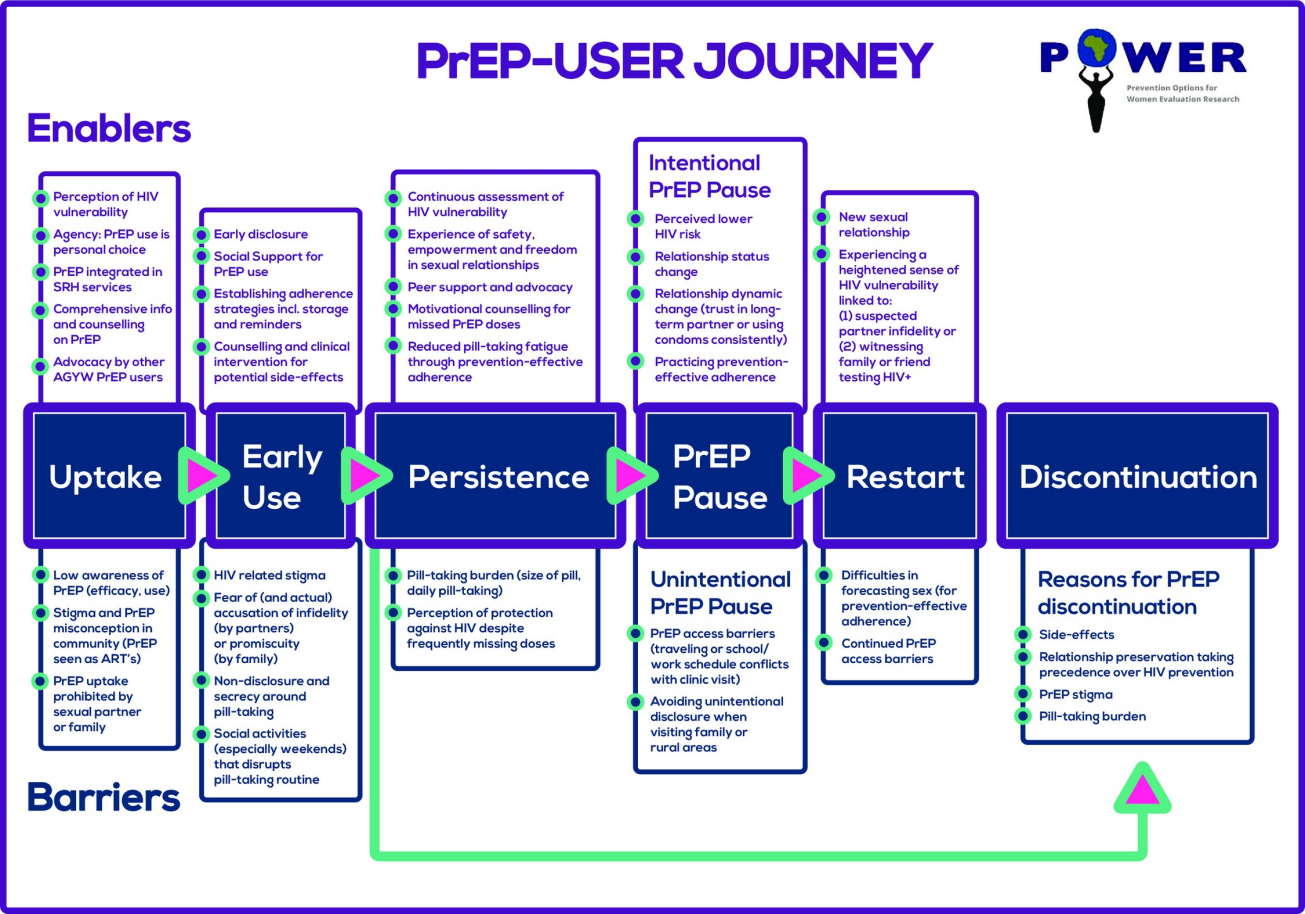
AGYW’s PrEP-user journey. Enablers and barriers at key stages in young women’s PrEP user journey from PrEP uptake to early use and persistence or discontinuation, including periods of PrEP pauses and restarts.

Qualitative results are described below according to key stages in young women’s PrEP-user journey (illustrated in Fig 1).

### AGYW’s considerations for PrEP uptake

AGYW had a number of considerations before deciding to initiate, delay or decline PrEP: (1) prior awareness about PrEP; (2) perceptions of their HIV vulnerability; (3) ease of PrEP access; and (4) stigma and PrEP misconceptions in the community.

#### Initial PrEP awareness among AGYW in their communities

PrEP awareness was very low in the communities where participants resided, with AGYW primarily hearing about PrEP for first time at the clinic where the POWER study took place, while seeking contraception or treatment for an existing STI.

“*Because I did the research about that [efficacy]*, *then they [staff at the clinic] told me it’s true that the pill works*, *so I feel safe that I’m taking the pill which is making myself to be safe from men that are taking advantage of young women”*. (IDI, 20-year-old, Johannesburg)

AGYW were generally enthusiastic to learn about PrEP and viewed it as something that young women have been waiting for.

“*[PrEP is] the breakthrough that we have been waiting for especially as a youth”* (IDI, 22-year-old, Johannesburg).

#### AGYW’s perception of HIV vulnerability

AGYW shared that knowledge of their vulnerability to HIV acquisition through a current sexual partner or sexual violence in their communities motivated them to consider PrEP uptake.

“*Well*, *every girl is at risk of HIV and every day because we live in a horrible world*, *where we are raped*, *we are exposed to a lot of bad stuff*, *so I felt PrEP was just the answer you know*, *just prevent a disease and not to worry so much about it*.*”* (IDI, 20-year-old, Johannesburg)

AGYW described their potential HIV exposure as due to their partners’ multiple concurrent partnerships, unknown HIV status, and low condom use (either to avoid hostile condom negotiations or to please a long-term partner). They also described PrEP as an attractive option for young women when they are susceptible to peer pressure, seduced by older men when under the influence of alcohol, or because they are engaging in transactional sex.

“*We always want to please our boyfriends or to please friends because of peer pressure*. *We engage in sex without using condoms*. *That is why I wanted to know more about PrEP”* (IDI, 19-year-old, Cape Town)

AGYW anticipated that their use of PrEP would provide a welcomed sense of safety, freedom and equalization of gender power in sexual encounters by enabling HIV prevention for AGYW independent from a male partner’s consent or cooperation.

#### Ease of PrEP access and informed decision-making processes

AGYW who initiated PrEP indicated that the ease and convenience of getting PrEP on the same day of their clinic visit and as part of an integrated sexual and reproductive health (SRH) service facilitated uptake. Acceptance of PrEP was further facilitated when AGYW received counseling and comprehensive information, in an easily understandable manner, while being afforded the agency to decide for themselves if PrEP was appropriate for them. Early initiators of PrEP did so without consulting their family or partners. They believed PrEP to be a personal decision and that their sexual health should take precedence over others’ opinions. They reported that making this decision for themselves boosted their self-esteem.

“*It has made me feel proud about myself*, *because it was my decision to take PrEP*…*based on my own needs and not other people’s [needs]—I saw that PrEP [is] good for me*.*”* (IDI, 19-year-old, Cape Town)

Nevertheless, AGYW shared how important peer support was in generating PrEP interest, including seeing peers using PrEP themselves or noticing the presence and advocacy of other AGYW PrEP users at the clinic.

#### Stigma and PrEP misconceptions in the community

Stigma and misconceptions among families, peers and communities played a role in AGYW delaying uptake or declining PrEP. This included HIV-related stigma conflating PrEP for prevention and ART for treatment, anecdotes that women who take PrEP are promiscuous, cheating on their partners, or sex workers. These concerns resulted in some young women first establishing approval from family or partners before initiation PrEP during a subsequent return to the clinic.

*The thing is*, *after that incident [hearing about PrEP from the clinic] I went to my mother and told her*. *And she said if I know it will help me*, *I should take it*. *Then I decided to come get it [the following day]*. (IDI, 16-year-old, Cape Town)

Conversely, for others, PrEP use was prohibited by sexual partners or family members and so they decided to not initiate.

*“He [boyfriend] asked me why I should use it*. *“Don’t you trust me*?*” he asked*. *“Do you have a side partner*?*” he continued*. *Those were the questions asked and I decided not to use it*.*”* (IDI, 21-year-old, Kisumu)

Other concerns or considerations mentioned prior to initiating PrEP were around efficacy and potential side-effects, the burden of daily pill-taking, and duration of PrEP use. Young women indicated that in some instances taking more time to consider PrEP’s relevance in their lives and learning more about PrEP’s benefits in the media or from peers, convinced them to come back to the clinic and start PrEP.

### Early PrEP use (0–3 months)

The early use phase was a period of adjustment for AGYW and included (1) disclosure; (2) seeking social support for PrEP use; and (3) thinking through pill-taking adherence strategies that suit their lifestyles.

#### Experiences of disclosure and social support

AGYW had a range of opinions and experiences regarding disclosure of their PrEP use to sexual partners, family, friends, and community. The benefits of disclosure after initiating PrEP were described as receiving social support for PrEP use and adherence reminders, which destigmatized taking PrEP in front of others. The primary deterrents and potential harms caused by disclosure included accusations of infidelity by partners; judgments about young women’s sexual lives and behavior; or stigma/misconception of PrEP as ART. AGYW practiced selective disclosure, deciding who they felt comfortable disclosing to as well as whose knowledge of their PrEP use would be valuable for their adherence. Young women who avoided disclosure described having to hide pills and take them in secret which made consistent use difficult.

PrEP use was primarily disclosed to family or the people with whom AGYW cohabited. Mothers’ support was of particular significance to AGYW and they appreciated when their parents encouraged them to make their own decisions about their health. Social support was described as beneficial to adherence and integrating PrEP into young women’s daily routines during early use.

“*It was supportive like at least there is someone who knows that I am taking them*, *I am not hiding this thing*. *Especially if my mother knows*, *then I am okay*. *About others and stuff*, *no*, *but just as long as my mother [knows]*.” (IDI, 18-year-old, Johannesburg)

While AGYW felt that disclosure to family would be beneficial, they had concerns about disclosing PrEP use to their sexual partner(s). The main apprehensions related to disclosure causing conflicts, decreased trust, or potentially causing a breakup of their intimate relationships.

“*My thoughts were that he [sexual partner] might not understand me and might think that I am sick [have HIV]*. *So*, *I was ashamed to tell him*.” (IDI, 20-year-old, Cape Town)

Participants stated that this fear of partners’ reactions caused young women to hide PrEP use or sometimes led to adjusting pill-taking times based on when they were meeting with a sexual partner. In some instances, initial negative reactions from family or sexual partners at disclosure changed to acceptance and support when more information about PrEP and AGYW’s reasons for use was provided to these significant others.

#### Early use adherence strategies and fitting PrEP into daily lives

AGYW shared strategies to integrate PrEP into their daily lives including pairing pill-taking with an existing daily activity (such as a television show or a specific meal); setting an alarm; or taking it with other medication such as contraception. A practical early use challenge was discreet PrEP storage, especially if a young woman was avoiding disclosure.

Missing pills at some stage during the PrEP-user journey was the norm and most missed doses were reported as unintentional due to unexpected circumstances or a disruption in their routine. Forgetting pills happened most frequently over weekends when AGYW were out with friends doing social activities, drinking alcohol, or staying over at a sexual partner’s place.

“*Ja*, *it [missing pills] happened*, *I had gone to a party [laughs] so I came back very late…I was just drunk and I had brought my friends home*. *I did not think about it [taking PrEP] that day*.*”* (IDI, 24-year-old, Johannesburg)

### Discontinuation

Fifty nine percent of the young women interviewed reported a PrEP interruption or discontinuation within the first 3 months of PrEP use. Reasons for PrEP discontinuation included (1) perceived side-effects and pill-taking burden, or (2) PrEP stigma and disapproval from family and sexual partners.

#### Perceived side-effects and pill-taking burden led to early discontinuation

Instances of discontinuation early in the PrEP-user journey occurred as a result of perceived side-effects (gastrointestinal, nausea, or headache) or the size of the pill, making swallowing challenging.

“*I changed my mind because of the side effects… and the size [of PrEP pill]*. *My mother and aunt have their medications that they are taking but out of all these medications my PrEP pill was the biggest one*… *and I didn’t feel well even when I was taking it*.” (IDI, 19-year-old, Cape Town)

Taking a pill daily for prevention was new to AGYW. The cognitive burden of daily pill taking and frequently forgetting doses discouraged continued PrEP use.

“*It was difficult for me*, *because I kept on forgetting [to take PrEP] I am not used in taking pills all the time*.*”* (IDI, 20-year-old, Cape Town)

“*Sometimes I just forget [to take PrEP]*, *I don’t know why… maybe because there will be no pain that will remind me that actually*, *it’s paining now*, *go and drink these tablets and stuff*.” (IDI, 18-year-old, Johannesburg)

#### Stigma and disapproval from family, peers and sexual partners

Young women described how despite initial motivation to use PrEP, they ended up discontinuing when they experienced stigma and a lack of social support.

“*The people in my life had the influence that PrEP is not something good*, *a lot of people*. *Like my mother because she is also against PrEP*. *So*, *as they think that PrEP is not good*, *I stopped PrEP*.*”* (IDI, 17-year-old, Cape Town)

In situations where AGYW experienced stigma from household members, maintaining a PrEP schedule was challenging as they attempted to take PrEP when they had privacy. This led to forgetting pills which was discouraging and sometimes concluded with discontinuation.

“*…cause every time I take them [PrEP pills]*, *my roommate used to ask me*, *‘why are you always taking pills’*, *she didn’t know anything about PrEP…And sometimes you will be embarrassed that maybe she would think that I am HIV positive or something*. *So that’s why I decided that hey let me rather stop taking them [PrEP pills]*.” (IDI, 19-year-old, Johannesburg)

The importance of relationship preservation for AGYW was also described by young women in the FGD’s, reflecting how HIV prevention decisions may be based on their anticipation of partners’ negative responses to PrEP (disapproval with or without the threat of violence).

“*Anyango stays with her husband who loves violence of which should he realize Anyango is using PrEP she will be badly beaten that day*. *He will accuse her of infidelity even though he is the one*. *So*, *she would not want the husband to know though it will be good if someone taught him about PrEP but Anyango herself can’t*.*”* (FGD, Kisumu)

Similarly, in South Africa a FGD participant indicated that Lebo may discontinue PrEP based *on “…pressure probably from her partner*. *He might say*, *‘if you are continuing [to take PrEP] sisi (sister)*, *then I request that we break up then”*. (FGD, Cape Town)

### PrEP persistence

Persistence was a phase in the PrEP-user journey that included creating personal and social strategies to remain motivated in taking pills consistently over a prolonged period of time, while anticipating and adapting to changes in one’s daily routine.

#### Sustaining motivation facilitates PrEP persistence

Sustained motivation for PrEP use was linked to AGYW continuously assessing and acknowledging HIV risk in their lives, along with forming clear intentions or reasons for PrEP use. AGYW shared how taking PrEP consistently allowed them to feel safe and more empowered, causing positive shifts in their relationship dynamics and sexual behaviour. Persistent PrEP users described increased comfort during sex without condoms and greater relational agency and communication with partners about HIV and dynamics around sex.

“*I feel relaxed because that risk of getting HIV I have controlled*.” (IDI, 19-year-old, Kisumu)

“*I just feel PrEP is really helping*, *so I am not afraid [of HIV]…It has made me feel so comfortable [with my partner]”*. (IDI, 17-year-old, Kisumu)

Young women, however, shared that taking PrEP could feel like a big responsibility and at times they needed to motivate themselves to overcome the burden of daily pill-taking. Pill-taking fatigue was lessened for AGYW through either practicing prevention-effective adherence or motivating themselves to continue PrEP use during their current season of perceived high HIV risk, knowing it is impermanent.

#### Barriers to persistence

Convenience of accessing clinics for PrEP refills facilitated AGYW’s continued engagement in SRH services, including PrEP persistence, while access barriers were frequently listed as reasons for PrEP interruptions. Young women shared how other obligations such as school and work often made it difficult to schedule their PrEP refill visits, leading to interrupted PrEP use and sometimes discontinuation. Participants in the FGD added that rumors about accessing PrEP from a local clinic also acted as a barrier to persistence.

“*People will think that she is sick [has HIV] if she keeps going to the clinic*, *to fetch treatment [PrEP perceived as ARV’s]*.*”* (FGD, Cape Town)

Non-persistors also seemed more affected by rumours and PrEP misconceptions and subsequently expressed doubts about PrEP’s efficacy. Paradoxically, this group of young women also mentioned feeling protected despite frequently missing PrEP doses. Conversely, AGYW who persisted shared the positive emotions (e.g. feeling empowered, confident and safe in sexual encounters) they experienced while adhering to daily PrEP taking, and negative emotions (e.g. guilt and stress over HIV exposure) resulting from skipping pills.

Non-persistors particularly encouraged more peer advocacy and suggested that more young women should use PrEP openly to de-stigmatize PrEP and build peer support for use. AGYW who struggled with persistence also expressed their preferences for smaller pills or a more discreet option like an injection that would solve a few of their challenges including not requiring home storage, avoiding issues around potential disclosure (no pills for partners or family to find), and less frequent clinic visits.

### PrEP pause and restart

Young women described pauses of varying length in PrEP use (1–9 months) as either intentional or unintentional.

#### Intentional PrEP pauses

AGYW decided to take a PrEP pause 1) during phases of perceived lower risk of HIV; 2) when practicing prevention-effective adherence in their sexual relationships; 3) to avoid unintentional PrEP disclosure; or 4) during times when PrEP use was perceived as difficult to fit into their daily lives (e.g. seasonal travelling). Whereas five participants were specifically interviewed due to their experience of restarting PrEP, many more spoke about pausing and restarting PrEP and their views were included in this section too.

AGYW shared that they perceived lower HIV risk and took a PrEP pause during relationship status changes (i.e., going through a breakup, not dating anyone, or when their partner is out of town) or changes in relationship dynamics, such as starting to trust a long-term partner or using condoms more consistently. AGYW also shared about practicing prevention-effective adherence when a sexual partner worked in another area.

“*You take it*, *after seven days that is when you can have sex with someone*. *This is because my partner is from [name of area] and whenever he is away*, *I can relax a bit [can pause PrEP use]*. *I will resume using it when he is coming back*. *He can tell me that he will come on such a day then it is up to me to plan myself for his coming*.”(IDI, 17-year-old, Kisumu)

PrEP pauses were also common when AGYW traveled to visit family or their rural homes as a means to avoid unintentional disclosure and stigma:

“*Sometimes maybe you visit [rural] home and then you don’t take them because you are afraid that there is a person who will see that you are taking the pills…you will find that they will spread the news wide*, *they will say you are sick [have AIDS]*, *all that stuff*. *You will no longer be appealing*.*”* (IDI, 25-year-old, Johannesburg)

Seasonal PrEP pauses were discussed by young women in the FGDs too. Specifically, AGYW in South Africa indicated that they would like to pause PrEP use over the December holidays, as this was a time of travel and partying, making it difficult to fit oral PrEP into their lifestyles.

“*Maybe in December*, *because it is the festive season*. *We have those ‘big days’… she will forget it [to take PrEP]*, *she is busy*. *During the festive season babe… it [taking PrEP] is just making things difficult for yourself*. *She won’t even be able to carry that thing [pillbox] of hers”* (FGD, Cape Town).

However, other young women in the same FGD group challenged seasonal PrEP pauses indicating that these were also seasons of heightened HIV risk and rather recommended a change in pill taking schedule.

“*I think the schedule [of when she takes PrEP pills] would change*. *I would take it in the morning*, *because in the morning you are home*, *things [parties/events] start late*. *She must not take it [the PrEP pause]*, *because she is not taking a break from sex*.*”* (FGD, Cape Town).

#### Unintentional PrEP pauses

AGYW experienced unintentional pauses primarily due to PrEP access barriers when traveling out of town; experiencing schedule conflicts (school or work commitments) with their PrEP refill visits; or when they lacked transport to a distant PrEP clinic.

“*I was in the village and couldn’t access it*. *I came back*, *and it took two months and a half [that she was off PrEP/away in the village)*. *I told my friend that we need to go back [to clinic] for the drugs*, *and that is how we came for refills*.*”* (IDI, 24-year-old, Kisumu)

AGYW had various perspectives on protection from HIV acquisition and PrEP efficacy during PrEP pauses. Participants typically did not seek out counseling before a PrEP pause and made their own assessments regarding prevention-effective adherence.

#### Restarting PrEP

PrEP restarts after a pause occurred when AGYW: 1) reconciled with a past sexual partner; 2) started a new relationship; 3) restored access to PrEP services; or 4) experienced a heightened sense of HIV vulnerability. These feelings of increased HIV vulnerability were described when AGYW witnessed a family member or friend testing HIV positive or due to a sexual partner’s behavior (e.g. instances of suspected multiple concurrent partnerships).

“*I paused [PrEP use] because my sexual partner agreed that we will be using condoms*, *but after sometimes*, *he refused [condom use] and I started again to use PrEP*.*”* (FGD, Kisumu)

AGYW, however, also described how despite understanding and practicing prevention-effective adherence, they sometimes experienced difficulties in forecasting sex, a drawback of this strategy. Young women in the FGDs agreed that Lebo/Ayango should return to the clinic for counseling on how to maintain daily PrEP use, or safely practice prevention-effective adherence as well as get tested for HIV and STI’s before resuming PrEP use.

### The role of counseling and healthcare providers in AGYW’s PrEP-user journey

AGYW emphasized the importance of adolescent-friendly, non-judgmental and supportive counselling at different stages during the PrEP-user journey. They highlighted that receiving clear and comprehensive information about PrEP, feeling supported by clinic staff and knowing what to expect (including side-effects) when taking PrEP were important and alleviated the initial concerns of AGYW. Young women shared that being able to ask questions about their SRH needs and intimate relationships to counsellors helped them prepare for potential use challenges and fitting PrEP into their daily lives.

*“It was great because they [clinic staff] clearly explained to me*, *and I was very clear about what this pill is and for what*, *and how it works*. *So*, *I was interested [in taking PrEP] because I understood what it was all about*.*”* (IDI, 19-year-old, Cape Town)

Young women also appreciated the short waiting times and discretion when PrEP was dispensed at the study clinics.

*“Coming to the clinic is not a big deal…it wasn’t going to consume my time a lot*. *The other thing that motivated me was that when I went to the pharmacy*, *the tablets were put in an envelope*. *That already no one can know what I am picking from the pharmacy*.*”* (IDI, 23-year-old, Kisumu)

During the early use phase, AGYW could discuss with counselors their decisions regarding disclosure, PrEP storage and adherence strategies. Counseling and support at the clinic also helped AGYW to navigate PrEP stigma and misconceptions circulating in the community. AGYW also reported feeling supported by clinic staff to persist with PrEP when they felt demotivated by family, peers or sexual partners, and counseling promoted a focus on prioritizing AGYW’s own health needs.

“*She [counsellor] gave me courage [to take PrEP] and made me realize that I’m doing this for myself and not anyone else*.” (IDI, 24-year-old, Johannesburg)

Young women who experienced side-effects after starting PrEP reported it to the clinic staff who provided reassurance that these are transient, and prescribed medications and advice to alleviate the symptoms of early PrEP use. Counseling about potential side-effects at PrEP uptake reassured young women that their bodies would adapt, which encouraged them to continue PrEP use.

During the PrEP early use and persistence phases counseling was focused on encouraging young women to continue taking PrEP despite missed doses; thinking through adherence strategies when traveling or when out with friends or sexual partners; as well as how to deal with lack of social support.

*“Yes*, *I told them [about missing doses]*. *They said there is no problem but next time I have to take all my pills*. *I didn’t feel bad because… she just said we are happy that you forgot once [to take PrEP] but next time we will be happy for you to take all of them [PrEP pills]*.*”* (IDI, 23-year-old, Johannesburg)

Young women in the FGDs viewed continued counselling and support as important for helping AGYW deal with adherence challenges and navigate PrEP restarts after a pause.

## Discussion

This qualitative study highlighted enablers and barriers for AGYW in SSA during their PrEP-user journey, from awareness and initiation to discontinuation or persistence. Daily oral PrEP use was shown to be challenging for AGYW due to individual (side-effects), relational (prioritizing relationship preservation), social (stigma, lack of support, and difficulty fitting pill-taking into lifestyle), and structural factors (access). We found that AGYW valued informed decision-making regarding PrEP uptake and prevention-effective adherence. PrEP interruptions (voluntarily or involuntarily) were part of the PrEP-user journey and AGYW exercised their judgment to decide about when to use, pause or discontinue PrEP. Disclosure, social support, non-judgmental adolescent-friendly counseling, and convenient access to PrEP were key to persistence.

PrEP uptake was facilitated through AGYW’s acknowledgement of HIV vulnerability in their lives and the benefits of being in control of HIV prevention in their relationships strongly influenced uptake and initiation. In addition, uptake was high when PrEP was offered as part of an integrated SRH service, but hampered by low awareness, stigma and PrEP misconceptions in the community. HIV prevention is embedded in social and interpersonal contexts [29–31] and AGYW in this study were found on a continuum from prioritizing their personal sexual health versus prioritizing others’ opinions [32]. This was evident during PrEP uptake where some AGYW embraced PrEP as a user-controlled HIV prevention method, while others were compelled to negotiate social acceptance through establishing PrEP use support from family or sexual partners before making this decision regarding their own sexual health. Research shows that AGYW’s agency, the sense of being in control of one’s own decisions, is highest in environments of opportunity (i.e., PrEP access, information and social support) and choice, but reduced when someone believes that their voluntary action will cause negative outcomes (to them or their relationships) [33]. We found that when strong personal agency was present in AGYW it positively affected the PrEP journey trajectory from uptake to persistence.

Early PrEP use (0–3 months) was an important period in the PrEP-user journey for establishing pill-taking strategies and seeking social support. Supportive and non-judgmental counseling was needed as a credible source for PrEP information at uptake and assisted AGYW during the early use phase to think through disclosure and dealing with potential early side-effects. Unfortunately, young women who experienced a lack of (perceived or actual) social support or PrEP-related stigma, avoided disclosure and subsequently struggled with persistence [18,34]. Similarly, AGYW whose sexual relationships were threatened by their PrEP use had more challenges with persistence, as reported in other research with female initiated methods [20,35–38]. In addition, AGYW need long-acting, discreet HIV prevention methods to reduce some of the barriers that daily pill-taking pose such as unintentional disclosure, cognitive and emotional burden, storage, and interference of competing life priorities such as having to fit the product into travels or weekend social life [39,40].

Persistence on PrEP was facilitated by AGYW’s continuous reevaluation of their HIV risk throughout the PrEP-user journey; family and peer support for PrEP use and reducing pill-taking fatigue through prevention-effective adherence. However, similar to previous research, for some AGYW, changes in relationship dynamics impacted their perception of HIV risk over time. Furthermore, the inability to accurately forecast sex complicated the practice of prevention-effective adherence [20,32,41,42]. In this study, convenient PrEP access within an integrated SRH, adolescent-friendly service supported both PrEP pauses and sometimes discontinuation. This underscores other PrEP demonstration studies which suggest that improvement in PrEP access is critical for AGYW’s PrEP journey [9,43–45]. PrEP use interruptions (both intentional and unintentional PrEP pauses) were also observed when young women had challenges fitting PrEP into their daily lives or when attempting to avoid unintentional PrEP disclosure to family or sexual partners [30,46]. While PrEP discontinuation was often a result of perceived initial side-effects, other factors like PrEP stigma, pill-taking burden, or relationship preservation also took precedence over AGYW’s desire for HIV prevention. This highlights the importance of approaching biomedical prevention as a strategy that must fit into users’ lives (and not the inverse). Research is needed to better understand pharmacological correlates of protection in AGYW like it has been established for men who have sex with men and transgender women [47–49]. If more foregiveness in oral PrEP adherence is demonstrated, this in turn may increase AGYW’s options and allow for pill-taking variations, for those of whom daily dosing is not working.

Findings from this study indicated that more work on counseling AGYW regarding prevention-effective adherence, PrEP pauses, restarts and persistence is necessary. We suggest that healthcare providers accept “seasons of risk” and normalize PrEP interruptions without shaming AGYW, create adherence and HIV protection feedback opportunities for AGYW, and create an environment where young women feel comfortable sharing the relational and social conditions related to their PrEP use or interruptions. The value young women placed on informed decision-making regarding PrEP uptake, pauses, and restarts should guide approaches to PrEP implementation. Under these conditions, AGYW can increase personal agency and effective PrEP use [50,51].

Our study had several limitations: it was qualitative and therefore inference regarding these findings cannot be applied outside of the study sample of AGYW and areas (urban and peri-urban areas in South Africa and Kenya). Our sample did not include participants who discontinued very early (before the month one clinic visit) and therefore our findings cannot be generalized to less engaged POWER study participants. The study, however, had a sizeable sample of 137 young women and triangulation was applied through gathering information from different data sources and data collection methods (IDI’s and FGD’s) in order to build a comprehensive understanding of AGYW’s PrEP-user journey [52]. Social desirability might be a factor in this study since participants knew that PrEP adherence was desired and therefor might have exaggerated their interest and commitment to PrEP use. Additionally, our qualitative findings should be considered in combination with the quantitative results of this study, which revealed high initiation with modest levels of persistence [27].

In summary, this study characterizes the enablers and barriers at each of the key moments in AGYW’s PrEP-user journey. We found that AGYW’s ability to exercise decisions regarding initiation and persistence with oral PrEP required convenient and integrated access to PrEP; clear, understandable and non-judgemental education, as well as personal and relational agency. These in turn helped AGYW carry out their choice to achieve the desired wellbeing outcome of feeling in control and safe from HIV infection. Young women desire comprehensive information on HIV prevention, and PrEP providers need to be aware of AGYW’s developing self-identity within a social context with limited support for their sexual health decisions. Our findings highlight that AGYW’s desire HIV prevention, but their PrEP-user journey is constrained by limited PrEP biomedical options (currently only daily oral PrEP) and service delivery models available to this population in SSA.

## Supporting information

S1 Appendix IDI guide. (DOCX)

S2 Appendix FGD thematic guide. (DOCX)

S3 Appendix Translated guides. (DOCX)
