# Supplementary material for: Adolescent girls and young women’s PrEP-user journey during an implementation science study in South Africa and Kenya
Source: PLoS One. 2021 Oct 14;16(10):e0258542. doi: 10.1371/journal.pone.0258542 (PMC8516266; doi:10.1371/journal.pone.0258542)
Supplement: S2 Appendix — (DOCX) [file pone.0258542.s002.docx]

**S2 Appendix. FGD thematic guide**

Focus group discussion (FGD) thematic guide in English and local language translations.

**Theme 1: PrEP Journey:** *To begin our conversation today, we’d like you to think about the following situation:*

***Lebo [or alternate name appropriate for specific location], a young woman living in your community [village/township], is thinking about whether she wants to use PrEP.***

*We’re now going to ask you various questions about Lebo and her PrEP user journey. Feel free to each choose different responses, there are no right or wrong answers. Use your responses to these first questions to help you think about what Lebo might do at each point in her PrEP user journey.*

**Let’s first describe who Lebo is…**

- - Does Lebo go to school and/or is she working? Who does she live with (husband/sex partner or parents/family members)?
  - How did she find out about PrEP in the first place? What did she hear? From whom?
    - Does she know if any of her friends are using PrEP? What do they say about it?

**Now let’s talk about what might come next after Lebo started thinking about whether or not to use PrEP.**

- - Why would she want to use PrEP? Why would she not want to use PrEP?
  - Did she talk to other people about the possibility of her taking PrEP? What do you think they would have said?
  - What does she need to consider before making her decision about whether to use PrEP?
    - Where would she find more information about PrEP?

**Lebo [or alternate name] decides she wants to use PrEP.**

- - What was the primary reason she decided to use PrEP now?
  - What concerns does she still have about PrEP?
  - Where does she go to get PrEP? (If she says the POWER clinic, what if the POWER clinic didn’t exist, where would she prefer to get PrEP?)
    - Why would she choose this place?
    - Any challenges with going there?
    - [If they haven’t already talked about their personal experience] What about for you personally, what was it like for you to make the decision to start using PrEP?

**Lebo [or alternate name] is now taking PrEP. Let’s talk about what that’s like for her.**

[General experience, Disclosure & Stigma:]

- - How would Lebo describe her experience of using PrEP?
    - What about taking PrEP does she find easy? Difficult? Please describe.
  - Does she tell other people that she is on PrEP? Who? Why?
    - How would they react? What would they say? What would they do?
    - Is she feeling judged? By whom?
  - Who does she decide (among her close friends/family/sex partner) **not** to tell about her PrEP use? Why?
  - How has your experience been different than Lebo’s?

[Acceptability & Adherence:]

- - How does she remember to take her pills? Does she always take them? Does she forget? What makes her forget?
    - What support might she need to use PrEP as instructed?
  - What is keeping her motivated to use PrEP?
    - Does she have any beneficial (positive) side effects from PrEP?
  - What challenges might come up?
    - Does she have any unwanted (negative) side effects?
    - What about unwanted social experiences (stigma, discrimination, partner abuse)?

How can she overcome those?

- - When would Lebo pause using PrEP? For how long? Why the break? How often does she take breaks?
  - [If haven’t already talked about their personal experience] What about for you personally, what would lead you to take a break from using PrEP?
  - Have you ever encountered stigma as a result of taking PrEP?
    - If yes, please describe the experience. Who was involved? What did they say? Where did this happen? How did it make you feel?

**Lebo stops using PrEP.**

- - How long had she been using PrEP?
  - What reasons or in what situations made her stop?
    - Were there other life situations that were more important to her than HIV prevention at that time?
  - Who influenced her decision?
  - How would she feel about herself after she stopped?
  - What difference is there between stopping and taking a break from PrEP?
  - [If haven’t already talked about their personal experience] What about for you personally, what would make you stop using PrEP?

**Lebo decides to start PrEP again.**

- - For how long was she off PrEP?
  - What would lead her to decide to take PrEP again?
    - Is this the same or different from what led her to take PrEP the first time?
  - What advice would you give her about re-starting PrEP?

**Thinking about your experience in POWER:**

- What other personal experiences have you had with PrEP that is similar or different than Lebo that you’d like to share?
- When this study ends, are you still interested in taking PrEP? Why or why not?
  - [If yes] How do you plan to continue accessing PrEP?

**Theme 2: Social Networks & Support Mapping:** *Next, we’d like you to learn more from you about the people who are most important to you regarding your PrEP journey:*

- I have given each of you a piece of paper with “me” in the center and three circles drawn around it since you are the center of your social world.
- Using the stickers provided to you (e.g. best friend, friend group, mother, father, other family, sex partner, clinic counselor, doctor/pharmacist, religious leader, clinic staff, BLANK stickers to add others) on the inner circle place the stickers of the people who you think **would have or have had** the **biggest influence (positive or negative)** on your ability to take PrEP for as long as you are interested in taking it, **regardless of whether you have told them about your PrEP use in real life or not**. You can place as many or as few (even none) as you’d like.
  - Write in any additional people who have been or would be influential of your PrEP journey who aren’t already covered by those listed on the stickers.
- Next, on the middle circle, place the stickers of the people who have or would have the next amount of influence.
- Finally, on the outer circle, place the stickers of the people who have or would have the least amount of influence on taking PrEP.

Now, **marking only the stickers for the people who HAVE HAD influence on your PrEP user journey:** Use the RED highlighter to highlight those who had a negative influence on your PrEP use. Use the BLUE highlighter to highlight those who had a positive influence. If someone was influential both positively and negatively, highlight with both colors.

Participant

[After everyone has finished placing their stickers] **Let’s start with the people you put in the inner circle.**

- Why are they the most influential? Are they supportive of your PrEP use? What have they said about it?
- What information do you think they need to be given in order to support your PrEP use?
  - Who should provide that information to them?
- What did you tell them? What would you like to tell them about your PrEP use that you haven’t already? How important is it to tell them about your PrEP use?
  - What would you want them to do or say about your PrEP use?

**Now let’s talk about those whom you placed in the middle circle.**

- Are they supportive of your PrEP use? What have they said about it?
- What information do you think they need or would need to support your PrEP use?
  - Who should provide that information to them?
- What did you tell them? What would you like to tell them that you haven’t already? How important is it to tell them about your PrEP use?
  - What would you want them to do or say about your PrEP use?

**Now let’s talk about those whom you placed in the outermost circle.**

- Are they supportive of your PrEP use? What have they said about it?
- What information do you think they need or would need to support your PrEP use?
  - Who should provide that information to them?
- What did you tell them? What would you like to tell them that you haven’t already? How important is it to tell them about your PrEP use?
  - What would you want them to do or say about your PrEP use?

**Theme 3: Wrap-up:** *Before we end our conversation***,**

- Is there anything else you’d like to share with me?
- Are there any questions you would like to ask me?

**Thank you all very much for participating in this discussion today.**
