# Supplementary material for: Adolescent girls and young women’s PrEP-user journey during an implementation science study in South Africa and Kenya
Source: PLoS One. 2021 Oct 14;16(10):e0258542. doi: 10.1371/journal.pone.0258542 (PMC8516266; doi:10.1371/journal.pone.0258542)
Supplement: S3 Appendix — (DOCX) [file pone.0258542.s003.docx]

**S3 Appendix. Translated guides**

**KiSwahili**

Mada 1: Maarifa ya PrEP [Kwa wanaokubli mwanzoni na wanaokataa]

Kwanza ningependa kusikia kuhusu jinsi ulivyo weza kufahamu kuhusu PrEP kwa kuanzia.

1. Ulisikia vipi kuhusu PrEP mwanzoni? Ni nini unajua kuhusu PrEP?
   1. Ulisikia wapi kuhusu au nani ambaye alikuambia kuhusu PrEP?
   2. Je, ulisikia chochote kuhusu PrEP katika vyombo vya habari? (Km. Redio, matangazo, mitandao ya kijamii?)
   3. Umesikia nini kuhusu PrEP?
2. Nani anaweza kuchukua PrEP na kwa nini?
3. Inachukuliwa kivipi?
4. Nini ilikupendeza kuhusu PrEP?
   1. Ni nini kilichokufanya kutaka kujua zaidi kuhusu PrEP?
5. Ni nini kilichokufanya kuja kliniki?
   1. Je kulikuwa na yeyote ambaye alikuhimitza kuja?

Mada 2: Uamuzi wa PrEP [Kwa wanaokubli mwanzoni /wanaokataa na wanaokubali baadaye]

Ningependa kuuliza kuhusu uamuzi wako wa awali kwa kutumia PrEP:

1. Uliamua kivipi kuwa kuchukua PrEP ni njema kwa ajili yako / si njema kwa ajili yako mwanzoni?
   1. Nini ilikuwa faida na hasara ulizingatia wakati ulikuwa ukifanya uamuzi?
   2. Je watu katika maisha yako waliathiri uamuzi wako kuchukua / kutochukua PrEP? (Kwa mfano mpenzi, familia, au marafiki)
   3. Ni hatari zipi za kiafya ulizingatia wakati wa kufanya uamuzi wako?
2. Hatari ya kupata virusi vya ukimwi
3. Hatari kutoka kwa kuchukua PrEP (madhara, matatizo ya afya ya muda mrefu)
   1. Jinsi gani mahitaji ya kupata PrEP na kuichukua mara kwa mara iliathiri uamuzi wako wa kama au kutoitumia?
4. ***[Kama swali hili halijaeleweka, toa mifano:]*** Kufikia PrEP, muda unaochukua katika kliniki; faragha; uwezo / nia ya kuchukua kila siku; kusahau, kuhifadhi tembe, uzito wa kutumia tembe, kupima virusi vya ukimwi
5. Je taarifa kutoka kwa wafanyakazi wa kliniki iliathiri uamuzi wako kuchukua PrEP kivipi?
   1. Ni nini washauri au mhudumu wa afya walikuambia?
   2. Je muliongea kuhusu wasiwasi zako kuhusu PrEP?

**Uamuzi wa *chombo* cha PrEP*:*** Hebu sasa tujadili kuhusu mpango wa kompyuta kibao, ambayo unaweza kuwa ulitumia wakati ulipokuwa ukusubiri kuonekana, ambayo sisi tunaiita “chombo cha uamuzi wa PrEP ".

1. Je, ulitumia chombo cha uamuzi? ***[Kama hakutumia chombo, ruka sehemu yote iliyosalia]***
2. Chombo cha uamuzi ulikuwa na manufaa kwa kukusaidia kufanya uamuzi kuhusu kuchukua au kutochukua PrEP?

Tafadhali nieleze

- 1. Hasa, ni sehemu gani katika chombo cha uamuzi kilicho kusaidia kuamua?
  2. Ni habari gani kutoka chombo cha uamuzi iliyokuwa muhimu kwa kukusaidia kuamua? Na kwa nini?

1. Nini inaweza kubadilishwa au kuboreshwa kwa chombo?

a. Maudhui au sehemu za chombo chenyewe**(*kama mshiriki ana shida kukumbuka sehemu za chombo, unaweza kumkumbusha hizi):***

1. 1.0-3.0 **sehemu za** **Ufunguzi**  (ikiwa na “Mbona sababu”)
2. 4.0 **Uchaguzi**: PrEP, kondomu, kupunguza wapenzi wangu wa ngono, Kujua kama mpenzi wangu ana virusi vya ukimwi, kama mpenzi wangu ana virusi vya ukimwi, anatumia madawa ya virusi vya ukimwi
3. 5.0 **PrEP dhidi ya PEP**
4. 6.0-11.1 **Sasa tuongee kuhusu PrEP**: Ni nini, Mbona kuchukua PrEP, Ni vipi vizuri inazuia virusi vya ukimwi, Natumia PrEP vipi, Nini unafaa kujua kuhusu PREP, Ukweli na uongo, nk.
5. 12.1 **Jua zaidi** – kufanya uchunguzi kuhusu ya kuzungumza na wafanyikazi wa kliniki
6. 13.0 **Upangaji uzazi**

b. Lini na wapi kinatumika

***[Kwa wanaokataa mwanzonii au akisema kuwa hakutumia PrEP]:***

1. Je, unaweza kufikiria hali ambayo unaweza kutaka kutumia PrEP? Kama ndiyo, tafadhali nieleze.

***[Kwa wanaokubali baadaye tu]:***

10. Nini ilifanya ubadilishe fikira yako na kuamua kuanza kutumia PrEP?

a. Je, uligundua taarifa yoyote, ikiwa ni pamoja na uvumi, ambayo ilifanya ubadilishe fikra yako kuhusu kutumia PrEP? Unaweza kuelezea?

b. Je watu katika maisha yako waliathiri uamuzi wako kuchukua / kutochukua PrEP? (Kwa mfano mpenzi, familia, au marafiki)

c. Je, unafikiri mabadiliko katika mawazo yako kuhusu kiwango chako cha hatari ilishawishi uchaguzi wako kuanza kutumia PrEP? Vipi?

- - 1. Hatari ya kupata virusi vya ukimwi
    2. Hatari kutoka kwa kuchukua PrEP (madhara, matatizo ya afya ya muda mrefu)

d. Je mabadiliko yoyote katika maisha yako iliifanya iwe rahisi kuchukua PrEP? (nyumbani, kazini, fedha, nyingine) Vipi?

- - 1. Je chochote kilifanyika katika kliniki ambayo ilifanya uamue kuanza kutumia PrEP? Niambie zaidi.

***[Kwa washiriki wote]***

**Mada 3: Uzoefu wako wa kliniki**

- - 1. Sasa ningependa kuzungumzia ziara zako za kliniki. Ilikuwa rahisi au ngumu kivipi kuja kliniki? Nini iliifanya rahisi au ngumu?
    2. Eleza ni nini huwa inafanyika wakati unaenda ziara yako ya kliniki kuchukua PrEP.
  1. Ilikuwa rahisi au vigumu kivipi kupata hudumana PrEP katika kliniki? Nini ilifanya iwe rahisi au vigumu?
  2. Kama umewahi ongeza dawa, ilikuwa vipi wakati ulienda kuongeza madawa? Ulienda wapi?
     1. Je, unaweza kuelezea ushauri na msaada ulipokea juu ya jinsi ya kuchukua PrEP?
  3. Ulielezwa nini?
  4. Ni nini ulipenda kuzihusu?
  5. Ni nini haukupenda kuzihusu?
  6. Je unahisi waliweza kushughulikia maswali zako na wasiwasi? Je unaweza nipa mfano
     1. Unafikiria ulihudumiwa kivipi katika ziara yako ya kliniki?
     2. Je, unaweza kunipa mfano wa kilichokuwa kizuri au mbaya?
     3. Je, unahisi kwamba umepokea ubaguzi wowote au hukumu kotoka kwa wafanyakazi wa kliniki? Kama ni hivyo, unaweza kutoa mfano?
     4. Ulijihisi huru kivipi kuzungumza kuhusu mada kama ngono, virusi vya ukimwi, PrEP, na mpango wa uzazi na wafanyikazi wa kliniki uliowasiliana nao wakati wa ziara yako ya POWER?
  7. Je unaweza kutoa mfano wa muda au wakati ulijisikia huru au kuwa na wasiwasi kuzungumza juu ya mada hizi? ni nini inaweza au ingekufanya ujihisi huru Zaidi?
  8. Ulijihisi huru kivipi ukiuuliza wafanyakazi wa utafiti maswali?

**Mada 4: Uzoefu wa kutumia PrEP *[Kwa mtu yeyote ambaye alichukua PrEP: waliokubali mwanzoni, waliokubali baadaye, wanaotumia vizuri, wasiyotumia vizuri na walio badilisha hali yao ya virusi vya ukimwi]***

Sasa ningependa kuzungumza kuhusu uzoefu wako wa kutumia PrEP.

- - 1. Kuchukua PrEP imekuwa vipi kwako?
       1. Nieleze kuhusu mara ya mwisho ulimeza tembe…ilikuwa lini, saa ngapi, ulikuwa unafanya nini, na ulikuwa wapi?
       2. Umekuwa ukihifadhi dawa wapi? Changamoto yoyote na kuhifadhi?
    2. Kuchukua PrEP imekufanya ujihisi kivipi kukuhusu?
  1. Umejihisi kivipi kimaumbile? (Madhara yoyoyte? Chochote cha manufaa)
  2. Umejihisi kivipi kimawazo?
     1. Ni mara ngapi unatumia PrEP?
  3. Je, ni mambo gani ambayo yamesaidia au kuifanya iwe rahisi kwako kutumia PrEP? Tafadhali eleza mikakati yoyote umetumia kukusaidia kutumia PrEP.
  4. Je, ni mambo gani ambayo imeifanya kuwa vigumu kwako kutumia PrEP?
     1. Jinsi gani umeshughulikia changamoto hizi?

1. Jinsi gani unafikiri umelindwa na nambari ya dozi uliyochukua?
2. Niambie kuhusu wakati umekosa kutumia kidonge chako cha PrEP.
   1. Nini ilikusababisha kukosa dozi?
   2. Je, ulizungumza na wafanyakazi wa kliniki kuhusu hilo? If no, why not? Kama la, kwa nini? Kama ndiyo, ni nini walisema? Je, ilikuwa na manufaa?
   3. Ni nini kinaweza kukusaidia kuzuia kukosa dozi tena?

***[Kwa wasiyoendelea kuitumia vizuri pekee]***

1. Kama uliwacha kutumia PrEP kwa muda wa kiasi chochote, au haukuchukua tembe ulizochukua, nini kilichokufanya kuacha? Probe:
   1. Je, ulipata taarifa yoyote, ikiwa ni pamoja na uvumi, iliyokufanya ubadili akili yako kuhusu kutumia PrEP? Unaweza kuelezea?
   2. Je, watu katika maisha yako waliathiri uamuzi wako kivipi ili uwache kutumia PrEP? (Kwa mfano, mpenzi, familia, au marafiki)
   3. Je, kulikuwa na mabadiliko yoyote katika maisha yako (nyumbani, kazi, mapato, na nyingine) ambayo iliifanya kuwa vigumu kutumia PrEP?
   4. Je, unafikiri mabadiliko katika mawazo yako kuhusu kiwango chako cha hatari ilishawishi chaguo wako wa kuacha kutumia PrEP? Kivipi?
      - 1. Hatari ya kupata Virusi Vya Ukimwi
        2. Hatari kutoka kwa kutumia PrEP (madhara, matatizo ya afya ya muda mrefu)
   5. Je, kutumia kidonge kila siku ilikuwa vigumu? Tafadhali eleza matatizo yako.
   6. Kuna sababu yeyote nyingine lililokusababisha kuacha kutumia PrEP?
2. Je chochote kilitokea wakati ulikuwa kwenye kliniki ambapo ilikufanya kuamua kuacha kutumia PrEP? Niambie zaidi.
3. Je, unafikiri utatumia katika siku za usoni? Kwa nini utatumia au hutatumia?

**Mada 5:** **Mahusiano**

1. Sasa hebu tujadili juu ya uhusiano wako. Je, kwa sasa uko katika uhusiano? (mpenzi mkuu, washirika wa kawaida)
   1. Niambie kuhusu mpenzi wako mkuu na vile uhusiano wenu uko.
   2. Je, una wapenzi wengine?
      1. Niambie kuhusu mahusiano yako na wao.
   3. Katika u(ma)husiano wako, je, umewahi kuongea kuhusu Virusi Vya Ukimwi na m(wa)penzi wako?
      1. ***[Kama ndiyo]*** Niambie zaidi juu ya mazungumzo umekuwa nazo.
      2. Umewahi kupima Virusi Vya Ukimwi pamoja na m(wa)penzi wako?
         1. ***[Kama ndiyo]*** Unajua hali ya virusi vya ukimwi ya m(wa)penzi wako?
   4. Je, umewahi kupitia aina yoyote ya vurugu katika uhusiano wako? (Viungo, hisia, ngono, kisaikolojia)
2. Je umeambia mtu mwingine kwamba unatumia PrEP au kwamba ulikuwa unafikiria kutumia PrEP? Mbona/mbona sivyo
   1. ***[Kama ndiyo]*** Ni nani uliambia? (Mpenzi, marafiki, familia) na hiyo ilienda aje?
      1. Je, unaweza kuniambia kuhusu jinsi watu katika maisha yako walivyojihisi kutokana na matumizi yako ya PrEP?
      2. Majibu ya nani ambaye yamekuwa muhimu zaidi?
      3. Nani amekuhamasisha katika kuchukua PrEP? Kivipi?
      4. Nani alikukatisha tamaa yako ya matumizi ya PrEP? Nini walisema?
      5. Je kuna mtu yeyote muhimu ambaye unamficha kutumia PrEP?
      6. Je kuna mtu yeyote muhimu ambaye haukumwambia? Mbona?
   2. ***[Kama hapana]*** Nini kilikufanya kuweka matumizi yako ya PrEP liwe siri?
3. Ni aina gani ya msaada au ushauri umepokea kutoka kwa wafanyakazi wa kliniki juu ya majadiliano kuhusu PrEP na m(wa)penzi wako/ familia?
   1. Walikuambia nini? Je walisema jinsi au kama ungemwambia mpenzi/ familia?
   2. Jinsi gani walikabiliana na changamoto uliyopitia?
      1. Ni katika njia gani msaada huo ulikuwa wa manufaa/ kutosha?
      2. Msaada huo ungeboreshwaje?
   3. Ni aina gani ya habari kuhusu PrEP ulikuwa nayo ili kumpa m(wa)penzi/ familia?
      1. Ni rasilimali gani nyingine ungependa kutoa au kushiriki na m(wa)penzi wako/ familia?

***[Kama aliwahi kutumia PrEP]:***

1. Je kutumia PrEP kimeathiri uhusiano wa familia yako yeyote? Vipi?
2. Je kutumia PrEP kimeathiri uhusiano wa ngono? Vipi? (km maamuzi, uwezeshaji, mawasiliano)
   1. Je na kuhusu utumizi wa kondomu na washirika wa ngono? Je hiyo imebadilika?

**Mada 6: Unyanyapaa na Ubaguzi**

1. Sasa ningependa kuzungumza kuhusu kile watu katika jamii yako wanasema kuhusu PrEP. Je, unaweza kuniambia kuhusu mambo yoyote wamesikia katika jamii yako kuhusu PrEP au utafiti huu? ***Hata hivyo hii ni kama amefafanua jamii*** ***-*** ***inaweza kuwa kitongoji, rika, nk]***
   1. Ni aina gani ya mawazo au uvumi umesikia zinazozunguka?
   2. Umesikia haya kutoka kwa nani?
   3. Je, unahisi kivipi kuhusu yale umesikia?
   4. ***[Kama si kweli]*** Una maoni gani kuhusu njia bora to address these ideas/rumors ? ya kushughulikia haya mawazo/ uvumi?
      1. Ni nani anafaa kushughulikia haya?
      2. Je, kuna chochote kliniki amefanya au ingefanya kushughulikia uvumi katika jamii au unyanyapaa?

**[Kama alitumia PrEP]:**

1. Je, unaweza kuniambia kuhusu uliyoyapitia ulipojihisi kuhukumiwa au kukoselewa na watu kwa kuwa huwa unafanya ngono auusing PrEP? kutumia PrEP?
   1. Ni nini wanasema?
   2. Nani anasema mambo hayo? Niambie zaidi kuhusu aina ya watu hawa. (mpenzi, familia, marafiki, jamii)
   3. Jinsi gani unhisi kuhusu kile wanachosema?
      1. Unafikiri kile wanachosema ni kweli au uongo? Kwa nini?

**Mada 7: Kubadili hali ya virusi vya ukimwi**  ***[Kwa wale ambao hali yao ya kutokuwa na virusi vya ukimwi imebadilika pekee]***

Kama ni sawa kwako, Ningependa kuzungumzia hali yako ya Virusi Vya Ukimwi.

1. Ni nini kwa maoni yako ilikuchangia kupata virusi vya ukimwi?
   1. Je, unaweza kuniambia zaidi kuhusu wakati fulani au tukio unafikiri unaweza kuwa umepata virusi vya ukimwi?
   2. Kabla ya kuambukizwa, je, kulikuwa na mabadiliko yoyote katika maisha yako binafsi, tabia, au hali ambayo iliweza kuongeza nafasi yako ya kupata virusi vya ukimwi?
      1. Mabadiliko katika uzingatiaji wa PrEP
      2. Mabadiliko katika tabia zingine za kuzuia
      3. Mabadiliko katiko mahusiano
   3. Jinsi gani unajisikia kuwa katika POWER imeathiri hatari yako ya kupata virusi vya ukimwi?
   4. Je, unahisi kuwa kwenye PrEP ingeathiri nafasi yako ya kupata virusi vya ukimwi?
2. Ni msaada gani ulipokea kutoka kwa wafanyakazi wa kliniki ulipopata matokeo yako ya kupimwa Virusi Vya Ukimwi?
   1. Je, ilikuwa yenye manufaa? Kama ndiyo, vipi? Kama siyo, ni jinsi gani inaweza boreshwa?
3. Niambie kuhusu unayoyapitia wakati wa kupata huduma za Virusi Vya Ukimwi.
   1. Umewahi pewa rufaa ili uende katika kliniki ya huduma ya virusi vya ukimwi?
   2. Je, umewahi kuwa katika kliniki ya huduma ya Virusi Vya Ukimwi?
      1. ***[Kama ndiyo]*** Njia gani wafanyakazi wa kliniki za PrEP walikupa msaada katika kwenda huko?
      2. Je, kwa sasa unatumia tiba ya kurefusha maisha?
      3. ***[Kama ndiyo]*** Ni gani uliyoyapitia ulipoanza tiba ya kurefusha maisha?

**Mada 8: Ushauri** ***[Kwa wanaoendelea kuitumia vizuri, wasiyoendelea kuitumia vizuri, na wale ambao hali yao ya kutokuwa na virusi vya ukimwi imebadilika ambao wamekuwa katika utafiti kwa muda wa miezi 3 au zaidi]***

Sasa ningependa kujifunza zaidi kuhusu yale umesema, au ungesema, kwa wanawake wengine vijana kuhusu PrEP.

1. Je umewahi washauri wanawake wengine vijana kuhusu PrEP?
   1. ***[kama ndiyo]*** Ni nani umezungumza naye? Sehemu zipi (km mashule, mitandao ya kijamii, vikundi vya jamii)
   2. ***[Kama la]*** Mbona sivyo?
2. Ni aina gani ya ushauri umepeana kwa wanawake vijana wengine kuhusu PrEP? ***[Mhoji: kama mshiriki hajawahi peana ushauri muulize kufikiria ni nini anaweza sema akiulizwa]***
   1. Anafaa kuichukua?
   2. Anahitaji kujua nini?
   3. Jinsi gani anafaa kutatua changamoto yoyote ambayo inaweza kuja wakati wa kutumia PrEP?
   4. Je, unaweza kumshauri ili awaambie watu wengine (mpenzi, familia, marafiki) kuhusu matumizi yake ya PrEP?
   5. Ni aina gani ya mabadiliko katika maisha yake anafaa kutarajia kama anatumia PrEP?
   6. Ni aina gani ya ushauri unaweza kumpa kuhusu kutumia kondomu wakati wa kutumia PrEP?

**Mada 9: PrEP katika siku zijazo** ***[Kwa wanaoendelea kuitumia vizuri, wasiyoendelea kuitumia vizuri, na wale ambao hali yao ya kutokuwa na virusi vya ukimwi imebadilika ambao wamekuwa katika utafiti kwa muda wa miezi 3 au zaidi]***

Sasa nataka tujadili juu ya vile utoaji wa PrEP inapaswa kuwa katika siku zijazo.

1. Ni mahali au mazingira gani itakuwa ni rahisi na salama kwenda na kupata PrEP?
   1. Uko tayari kusafiri umbali gani kwenda kupata PrEP?
   2. PrEP kwenye kliniki?
   3. Ni maeneo gani na masaa itakuwa rahisi zaidi ya kupata PrEP?
2. Jinsi gani yale unayoyapitia kwenye kliniki yanaweza fanywa kuwa bora?
   1. Zinaweza fanywa kuwa bora?
   2. Jinsi gani nafasi ya kliniki au mazingira inaweza fanywa kuwa bora?
   3. za kuongezwa dawa inaweza fanywa ili iwe nzuri zaidi?
3. Ni aina gani ya mtoa huduma ungependelea kwenda kwake kuchukua maagizo ya PrEP na kuongezwa dawa?
   1. Tafadhali nieleze unayoyapitia kwenye kliniki ambapo ulijisikia vizuri na mtoa huduma ***[Ili*** ***kuamua tabia ya watoa huduma ambao anahisi kuwa na starehe nao]***
   2. wafanyakazi wa kliniki ili unavyoonekana liwe nzuri zaidi?
4. Wafanyikazi wa kliniki wanawezaje kuwasaidia wanawake vijana kuhifadhi PrEP kiusalama na faragha nyumbani?
5. Ni aina gani ya habari au ushauri unafikiri inaweza kuwa ya manufaa kwa wanawake vijana ambao wanataka kutumia PrEP?
6. Ni aina gani ya msaada ya kijamii (kutoka kwa mpenzi, marafiki, familia) wanawake vijana wanahitaji kuwasaidia ili watumie PrEP?

***[All participants]*** ***[Washiriki wote]***

**Mada 10: Tunapomaliza**

1. Je, una mawazo yoyote mengine au maswali? ***[Andika chini*** ***maswali*** ***na uhakikishe*** ***umeyajibu au mpe mshiriki rufaa kama inavyohitajika].***

Maswali mengine yeyote tungepaswa kuuliza kuhusu namna ya kuboresha utoaji wa PrEP au urahisi wa kuitumia?
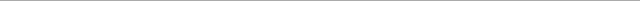


**Dholuo**

**Kidieny 1: Ng’eyo ewi PrEP *[Ne jogo moyie tiyogo e chakruok kod jogo motamore]***

1. Ere kaka ne iwinjo ewi PrEP mokuongo? En ang’o ming’eyo ewi PrEP?
   1. Ne en Kanye mane iwinje ewi kata ng’ama ne onyisi ewi PrEP?
   2. Bende ne iwinjo gimoroamora ewi PreP eyor fwambo (kaka. redio, lendo moyiedhi, yore fwambo mag oganda?)
   3. En ang’o misewinjo ewi PrEP?
      1. En ng’a manyalo kawo PrEP to nang’o?
      2. Ere kaka ikawe?
2. En ang’o mane olombi ewi PrEP?
   1. En ang’o mane omiyo igombo ng’eyo mang’eny ewi PrEP?
3. En ang’o mane ojiwi mondo ibi kar thieth?
   1. Bende nitiere ng’ama ne ojiwi mondo ibi kar thieth?

**Kidieny 2: Timo yiero ewi PrEP *[Ne jogo moyie tiyogo e chakruok /jogo motamore kod jogo moyie tiyogo bang’e]***

Daher penji ewi yiero mari tiyogi PrEP e chakruok:

1. Ere kaka niyiero ni kawo PrEP ne ber ne in/okber ne in e chakruok?
   1. Ang’o gini maber kata marach mane ing’iyo kane ikawo yiero mari?
   2. Ere kaka jogo mantie e ngimani ne ochocho yiero mari e kawo/kik ikawm PrEP? (kaka. jahera, anyuola, kata osiepe)
   3. Gin weche mage machandi mag thieth mane ing’iyo kane ikawo yiero mari?
      1. Weche machandi mar gamo kute mag ayaki
      2. Weche machandi kuom kawo PrEP (rach manyalo betie, gigo motenore gi gi weche thieth mayware)
   4. Ere kaka gigo madwarore mar yudo PrEP kod kawe kinde ka kinde chocho yiero mari e kaka kata koso tiyogo?
2. ***[Kapenjoni okwinjre maler, chiw ranyisi:]*** Yudo PrEP, thuolo mokaw e kar thieth, Kama okoyangore; nyalo/gombo kawo pile; wichwil; Kano andila, Pek mar kawo andila, pimo kute mag ayaki
3. Ere kaka wach mowuok kuom jotij klinik chocho yiero mari kawo/kik ikaw PrEP?
   1. En ang’o mane Johocho kata jochiw thieth ne ontisi?
   2. Bende ne uloso ewi weche machandi ewi PrEP?

**Gir timo yiero ewi PrEP*:*** Koro watwage ewi program mar tablet/Ipad ma samoro itiyogo kapod irito thuolo mari ma waluongo ni “PrEP decision tool”.

1. Bende ne itiyo kod gir yiero? ***[Ka ne okotiyogo gi gir yiero, chikri kidieny moluwo no tee]***
2. Ere kaka gir yiero nen gi kony e konyi kawo yiero mari ewi kawo/ kata ok kawo PrEP?

Yie iler:

- 1. Ahinya hinya, e Kanye e gino mane okonyi ng’ado rieko?
  2. En wach mane e gino mane ikonyi ahinya e ng’ado rieko? Nang’o?

1. Ang’o minyalo lok kata ket maber e gir yiero?
2. Kidienje mar gir timo yiero owuon kata puonj mane iye ***(ka jachiwri yudo matek e paro gima nitiere e rie yierono, inyalo parone magi)***:
   1. 1.0-3.0 **Michako godo** (riw “Nang’o”)
   2. 4.0 **Yore manitiere**: PrEP, rabo yunga, duoko piny namba mar johera mag ringruok achiel, ng’eyo ka jaherana nigi kute mag ayaki, ka jaherana ni kute mag ayaki, otiyo kod yeth
   3. 5.0 **PrEP ka ipimo kod PEP**
   4. 6.0-11.1 **Walos ewi PrEP** menu: En ang’o, nang’o itiyo kode, ere kaka ogeng’o gamo kute mag ayaki, gima onego ing’e ewi PrEP, adier kod miriambo, kod gik ma kamago.
   5. 12.1 **Somo ma omedore** – yiero gigo ma onego ilose gi jatij klinik
   6. 13.0 **Komo nyuol**
3. Karang’o gi kanye ma itiye kode

***[Mar jogo mane otamore e chakruok kata owacho ni oknotiyo kod PrEP]:***

1. Bende inyalo paro e kinde minyalo dawaro tiyog kod PrEP? Ka ee, kiyie lerna.

***[Mar jogo mane oyie bang’e kende]:***

1. Ang’o mane omiyo iloko pachi kendo yiero chako tiyo kod PrEP?
   1. Bende nifwenyo wach moroamora, koriwo gi fwambo, mane omiyo ilok pachi ewi tiyo kod PrEP? Bende inyalo lero?
   2. Ere kaka jogo mantie e ngimani ne nyalo siro yiero mari eloko pachi ewi tiyo kod PrEP? (kaka. Jahera, anyuola, kata osiepe)
   3. Bende iparo ni lokruok e pachi ewi thuolo mari rach ne siro yiero mari mar chako tiyo kod PrEP? Nade?
      1. Rach mar gamo kute mag ayaki
      2. Rach kotenore gikawo (rach manyalo betie, side effects, gigo motenore gi gi weche thieth mayware)
   4. Bende lokruok moro amora engimani nokete mayot kawo PrEP? (odu, tich, yor pesa, mamoko) Nade?
2. Bende gimora mora notimore ekar thieth mane omiyi yiero Chako tiyo kod PrEP? Nyisa mathoth.

***[Mar jochiwre duto tee]:***

**Kidieny 3: Lony ni mar kar thieth**

1. Koro daher loso ewi wuoth mari mar kar thieth. Ne yot kata tek marom nade biro e klinik? En ang’o mane okete mayot kata matek?
2. Ler gimatimore ekinde midhi kar thieth sama idhi e limbegi mag PrEP?
3. Ere kaka ne yot kata tek yudo kony mag thieth kod PrEP e klinik? En ang’o mane okete mayot kata matek?
4. Ka osemedi yath, ne en nade mane idhi mondo omedi yath? Ne idhi Kanye?
5. Bende inyalo lero hocho to kod kony mane iyudo kotenore gi kaka itiyo kod PrEP?
6. Ango mane onyisi?
7. Ang’o gini name ihero?
8. Ang’o gini mane okiher?
9. Bende iparo ni negi duoko penjogi kata weche manechandi? Bende inyalo miya ranyisi
10. Iparo ni ne oneni nade chieng limboni ekar thieth?
11. Bende inyalo miya ranyisi mar gima ne ber kata rach?
12. Be ipani iseyudo akwede kata jaro kowuok kuom jatich ekar thieth? Ponono, be inyalo miya ranyisi?
13. Ne in thuolo marom nade kod jatij klinik kapenji penjo ewi weche kaka bedo e achiel kuom ringruok, kuta mag ayaki, PrEP to kod komo nyuol ndalo limbegi mag POWER?
    1. Bende inyalo miya ranyisi mar kinde mane isebet thuolo kata ma pok ibet thuolo loso ewi weche gi? En ang’o maketi/ nyalo keti imed bed thuolo?
    2. Ne in thuolo marom nade chiwo penjo ne jatij nonro?

**Kidieny 4: Lony ewi tiyo gi PrEP *[Kuom Jogo duto mane otiyo kod jogo mane ochake echakruok, jogo mano chake bang’e, jogo manetiyokode maber, jogo mane okti kode maber kod jogo machal mar gi mar kute mag ayaki olokore]***

Koro daher mar wuoyo kodi ewi lony ma in godo ewi tiyo kod PrEP?

1. Tiyo kod PrEP iseneno kachal nade ne in iwuon?
   1. Nyisa ewi chieng mogik mane imuonye yath…ne en chieng mane, ne en saa adiwa, ne itimo ang’o, to kendo ne in kanye?
   2. Isebedoga ka ikano yath Kanye? Bende nitiere chandruok moro amora e keno?
2. Ere kaka muonyo PrEP osetimo iparo kuomi?
   1. Isewinjo nade e dendi in iwuon( Bende nitiere rach ma okelo e dendi? To bende nitiere ber ma okelo)
   2. Isewinjo nade e chunyi?
3. Ikawo PrEP mang’eny machalo nade?
4. Gin ang’o gini mosekonyi/kete mayot ni kawo PrEP? Kiyie lerna yore moro amora misetiyogo e konyi kawo PrEP.
5. Gin ang’o gini mosekete matek ne in kawo PrEP?
   - 1. Ere kaka isekedo kod pek gi?
6. Ere kaka iparo maber ni iritori kod # mar yath ma isekawo?
7. Kona ewi kinde mane ibari kawo andila mar PrEP.
8. Ang’o mane omiyo ibari kawo yath?
9. Bende ne iloso kod jatich ekar thieth ewach ni? Ka ooyo, ang’o momiyo? Ka ee, negiwache ang’o? Bende ne okonyi?
10. Ang’o manyalo konyi geng’o mari kawo yath kendo?

***[Ne Jogo mane ok ti gi yath maber kata jogo ma tiyo godo maber to olero ni ne nitie chandruok mar koso muonyo]***

1. Kane iweyo tiyo gi yadh PrEP e kinde moro amora, kata ne ok imuonyo yedhe mane ikawo, ang’o ma omiyo ne iweyo?
   1. Bende nifwenyo wach moroamora, koriwo gi fwambo, mane omiyo ilok pachi ewi tiyo kod PrEP? Bende inyalo lero?
   2. Ere kaka jogo mantie e ngimani ne nyalo siro yiero mari eloko pachi ewi tiyo kod PrEP? (kaka. Jahera, anyuola, kata osiepe)
   3. Bende ne nitiere pek e ngimani (e odi, kar tich, yuto, mamoko) mane okete matek tiyo kod yadh PrEP?
   4. Bende iparo ni lokruok e pachi ewi thuolo mari rach ne siro yiero mari mar chako tiyo kod PrEP? Nade?
      1. Rach mar gamo kute mag ayaki
      2. Rach kotenore gikawo (rach manyalo betie, side effects, gigo motenore gi gi weche thieth mayware)
   5. Bende muonyo andila pilepile ne tek? Yie iler pek go.
   6. Bende nitie gigo mamoko mane itimo iweyo tiyo kod yadh PrEP?
2. Bende gimora mora notimore ekar thieth mane omiyi iweyo tiyo kod PrEP? Nyisa mathoth
3. Bende iparo ni ibiro tiyo kod PrEP e kinde mabiro? Nang’o ibiro tiyo godo/ ok ibi tiyo godo?

**Kidieny 5:** **Tudruok**

1. Koro walose weche tudruok magi. Bende intiere tudruok moro amora gi sani? (Jahera maduong, Johera ma wiye wiye)
   1. Kona ewi jaherani maduong kod kaka tudruok maru chal.
   2. Bende in kod johera mamoko?
      1. Kona ewi tudruok mari kodgi.
   3. E tudruok ma intiere, bende isewuoyo ewi kute mag ayaki kod joheragi/ jaherani?
2. ***[Ka ee]*** Kona matut ewi twak ma usebedo godo.
3. Bende usebedo e pim mar kute mag ayaki kanyakla kod jaherani/ joherani?
   1. ***[Ka ee]*** Bende ing’eyo chal mar pim mar kute mag ayaki mar jaherani/ joherani?
   2. Bende isekadhe kit tulo moro amora? (lweny, midhiero, yor terruok, kor ka paro)
4. Bende isekono ng’at ang’ata ni itiyo kod yadh PrEP kata ni iparo tiyo kod yadh PrEP?
5. ***[Ka ee]*** Isekono ng’a? (Jahera, osiepe, anyuola)
6. Inyalo kona kaka jii e ngimani paro ka oluwore gi tiyo kod PrEP?
7. Ng’awa ma pache osebedo gi pek?
8. Ng’awa mane ojiwi ewi tiyo kod PrEP? Nade?
9. Ng’awa mane ochido chunyi kuom tiyo kod PrEP? En ang’o mane giwacho?
10. Bende nitiere ng’at ma igeno ma ipandone? Nang’o?
    - - 1. Bende nitiere ng’at ma igeno mane okinyiso? Nang’o
11. ***[Ka ooyo]*** En ang’o mane omiyo iparo keto tiyo mari kod PrEP malingling?
12. En kony kata paro mane ma iseyudo ma owuok kuom yachiw thieth ewi wuoyo kod jaherani/ anyuola ewi PrEP?
13. En ang’o mane gi nyisi? Bende negi nyisi kaka koso ka onego ikon jahera/ anyuola?
14. Ere kaka negi timo ewi chandruok mane iyudo?
15. Konyno ne ber e yoo mane/ bende ne konyno oromo?
16. Ere kaka konyno inyalo los mondo omed bedo maber?
17. En wach mane ewi PrEP mane in gido mondo imi jaherani/ anyuola?
18. Gin gigo mage ma inyalo gombo mondo imi kata inyis jaherani/ anyuola?

***[Kane otiyo gi PrEP kata matin]:***

1. Bende tiyo kod PrEP isekelo lokruok moro amora e anyuolani? Nade?
2. Bende tiyo kod PrEP isekelo lokruok moro amora e tudruok mari mar ringruok achiel? Nade? (kaka ng’ado rieko, tiego mar tego, tudruok)
3. To tiyo kod rabo yunga kod joheragi? Bende oselokore?

**Kidieny 6: Akwede gi ajara**

1. Koro adwaro ni walose gima ji e oganda wacho kuom PrEP. Bende inyalo nyisa gigo ma ji wacho e oganda kuom PrEP kata nonroni? ***[Kata kamano ma en gima oparo ni oganda en – nyalo bedo kama odake, jogo ma rom kode, kod gik ma kamago]***
   1. Gin paro mage kod fwambo ma isewinjo ka landore?
   2. Ne iwinjo magi ka iwacho gi ng’awa?
   3. Ere kaka iwinjo ka oluwore gi gima ne isewinjo?
   4. ***[Ka miriambo]*** En yoo mane ma iparo ni ber mondo olos godo paro/ fwambo gi?
      1. Ng’awa ma onego olos gigi?
      2. Bende nitiere gima klinik osetimo kata nyalo timo mondo olos fwambo kod akwede mane oganda?

**[Kane otiyo kod PrEP]:**

1. Inyalo nyisa kaka isewinjo ka iparoni ing’adoni bure kata ipingo gi jii nikech in ng’at ma osebedo e ringruok achiel kata ni itiyo kod PrEP?
   1. En na’go ma giwacho?
   2. Ng’awa ma wacho gigo? Kona mathoth kuom jogi. (Jahera, anyuola, Osiepe, oganda)
   3. Iwinjo nade kuom gima giwacho?
      1. Bende iparo ni gima giwacho en adiera koso mirambo? Nang’o?

**Kidieny 7: Lokruok mar chal mar kute mag ayaki** *[****jogo machal margi mar kute mag ayaki olokore kende]***

Ka ber kodi, agombo mar wuoyo ewi chal mari mar kute mag ayaki.

1. En ang’o e pachi mane omiyo igamo kute mag ayaki?
   1. Bende inyalo nyisa saa kata gima ne timore ma iparo ni ne igame kute mag ayaki?
   2. Kane pok igamo kute mag ayaki, bende ne iwinjo lokruok e ngimani, timbegi, kata gima ne omedo nyalo mar yudo kute mag ayaki?
      1. Lokruok kuom tiyo gi PrEP kaka dwarore
      2. Lokruok e timbe ma moko mag geng’oruok
      3. Lokruok e yor tudruok
   3. Ere kaka iparo ni Bedoni e POWER omulo nyalo mari mar yudo kute mag ayaki?
   4. Bende iparo ni Bedoni ka itiyo kod PrEP omedo koso oduoko piny nyalo mari mar gamo kute mag ayaki?
2. En kony mane mane iyudo ka owuok kuom jatij klinik kane iyudo duoko mar chalni mar kute mag ayaki?
3. Bende ne okonyi?? Ka ee, nade? Ka ooyot, ere kaka inyalo kete omed bedo maber?
4. Kona kaka osebedoni e yudo thieth mar kute mag ayaki.
5. Bende osekoni ni idhi e klinik kama iyude thieth mar kute mag ayaki?
6. Bende isedhiga e klinik ma ichiwe thieth mar kute mag ayaki?
   - 1. ***[Ka ee]*** Ere kaka jatij klinik e weche PrEP ne okonyi e dhi kuro?
     2. Sani bende intiere e thieth mar kute mag ayaki?
     3. ***[Ka ee]*** Ne ochalo nade kane idonje thieth mar kute mag ayaki?

**Kidieny 8: Ng’ado rieko** *[****Ne jogo mane tiyo kode maber, jogo mane okti kode maber kod jogo machal margi mar kute mag ayaki ne olokore to osebedo e nonro kuom dweche adek kata mang’eny ne****]*

Koro adwa ng’eyo mathoth kuom gima isewacho, kata ne igombo wacho, ne mine moko matindo ewi PrEP.

1. Bende isekonyo mine moko matindo ng’ado rieko ewi tiyo gi PrEP?
   1. ***[ka ee]*** En ng’awa mane iseloso godo? Ne un Kanye? (kaka e skul, yore fwambo manie oganda, grube manie oganda)
   2. ***[ka ooyo]*** Nang’o podi?
2. En rieko mage mane ing’ado kata inyalo ng’ado ne nyako moro matin ewi PrEP? ***[Jatim nonro: ka jachiwre pok okonyo ng’ato ng’ado rieko, nyise otem paro gima onyalo wacho ka diponi openje.]***
3. Bende onego oti kode?
4. En ang’o ma onego ong’e?
5. Ere kaka onyalo timo sama chandruok nitiere ka oluwore kod tiyo kod PrEP?
6. Bende inyalo ng’ado ne rieko mondo okon joma moko (jahera, anyuola, Osiepe) ni otiyo kod PrEP?
7. Gin chandruok mage e ngimane ma onego ogen ni nyalo bedo ka otiyo kod PrEP?
8. En pare mane ma inyalo miye ewi tiyo gi rabo yunga sama otiyo kod PrEP?

**Kidieny 9: PrEP e ndalo mabiro** *[****Kuom jogo manetiyokode maber, jogo mane okti kode maber kod jogo machal margi mar kute mag ayaki olokore to osebedo e nonro e kind dweche adek kata mang’eny****]*

Koro adwaro ni walose kaka chiwo PrEP onego obed e ndalo mabiro.

1. En Kanye ma nyalo bet ka yot kendo ber mondo ng’ato odhiye mondo omiye yadh PrEP?
   1. Diher mar dhi mabor madirom nade mondo iyud PrEP?
   2. En saa madirom nade ma nyalo bedo maber ka ng’ado rito mondo onene chieng chiwo PrEP?
   3. En Kanye kod saa ma nyalo bedo maber ahinya mar yudo PrEP?
2. Ere kaka inyalo ket kaka ineno ji e klinik mondo obed maber?
3. Ere kaka gigo ma itimo e klinik sama ineno ji inyalo ket mondo obed maber?
4. Ere kaka nafas mane klinik inyalo ket mondo obed maber?
5. Ere kaka yore mag medo ji yath inyalo ket mondo obed maber?
6. En jachiew thieth mane ma dine igomb dhi ire mondo ondikni yadh PrEP kendo omedi ka orumo?
7. Yie mondo ilerna kaka ne oneni gi jachiew thieth ma iwinjo ka in thuolo kode ***[Ma wadwa ng’eyo godo kit jachiw thieth mane en thuolo godo]***
8. En ang’o ma inyalo loko kuom jachiw thieth mondo iwinj ka in thuolo sama ineni?
9. Ere kaka jotij klinik nyalo konyo mine matindo kano PrEP kama ber kendo ok oyangore e ot?
10. En wach machalo nade kata hocho ma iparo ni nyalo konyo mine matindo madwaro muonyo PrEP?
11. En kony mane ma wuok kuom oganda (ka owuok kuom jahera, osiepe, anyuola) ma nyiri matindo dwaro mondo okonygi muonyo PrEP?

***[Ne Jo chiwre duto tee]***

**Kidieny 10: Ka watieko**

1. Bende in kod paro kata penjo mamoko? ***[Ndik gi piny kendo iduokgi kata iore ir kama onyalo yudo kony kaka dwarore].***
2. Bende nitiere penjo mamoko mane onego wapenj ewi kaka wanyalo medo loso yore mag chiwo PrEP kata kaka inyalo kete mayot tiyo godo?

**Zulu**

**Indikimba 1: Ulwazi nge-PrEP [Okwalabo okwathi zisuka bavuma nabanqaba]**

1. Wezwa kanjani nge-PrEP okokuqala ? Wazini nge-PrEP?

a. Uzwephi ngayo noma ubani okutshele nge-PrEP?

b. Kungabe kukhona okuthile okuzwile nge-PrEP kwezezindaba (isib. umsakazo, ngokokwenza kwaziwe, ngezokuxhumana zomphakathi

c. Uzweni nge-PrEP?

i. Ubani ongaphuza i-PrEP futhi ngobani?

ii. Iphuzwa kanjani?

2. Yini eyakuchaza nge-PrEP?

a. Yini eyakwenza wafuna ukwazi kabanzi nge-PrEP?

3. Yini eyakugqugquzela ukuba uze ekliniki?

a. Kungabe kukhona othile owakugqugquzela ukuba uze?

**Indikimba 2: Isinqumo sePrEP [Okwabavuma/banqaba zisuka, nabavuma sekudlule isikhathi]** Ngizothanda ukukubuza ngesinqumo sokuqala mayelana ne-PrEP:

4. Wanquma kanjani ekuqaleni ukuba ukuphuza i-PrEP kwakukuhle kuwe noma kwakungekuhle?

a. Kwakuyini okuhle nokubi owakucabangisisa ngesikhathi uthatha isinqumo?

b. Kungabe abantu abasempilweni yakho basithinta kanjani isinqumo sakho sokuphuza noma ukungaphuzi i-PrEP? (isib. umaqondana, umndeni, noma abangani)

c. Ikhona kuphi kwezempilo okwakukukhathaza owakucabangisisa ngesikhathi uthatha isinqumo sakho?

i. Ukukhathazeka noma ubungozi bokuthola igciwane lengculaza

ii. Ukukhathazeka ngokuphuza i-PrEP ( imithelela engemihle, imithelela yezempilo ngokuhamba kwesikhathi)

d. Okwakudingeka ukuze uthole i-PrEP nokuyiphuza njalo kwasithinta kanjani isinqumo sakho sokuyisebenzisa noma sokungayisebenzisi?

i. [Uma lombuzo ungacacile, nikezela ngezibonelo:] Ukufinyelela kwiPrEP, isikhathi esichithwa ekliniki, ingasese, ukukwazi/ukuthanda ukuyiphuza nsuku-zonke, ukukhohlwa, ukugcinwa kwephilisi, umthwalo wephilisi, ukuhlolela igciwane lengculaza

5. Kungabe imininingwane evela kwabasebenza ekliniki isithinte kanjani isinqumo sakho nge-PrEP?

a. Bakutshelani abeluleki noma onikezela usizo ngezempilo?

b. Kungabe wakhuluma nganoma yini eyayikukhathaza nge-PrEP? Ithuluzi Lokuzinqumela lePrEP: Ake sixoxisane ngohlelo lwephilisi/ lweIpad okungenzeka ukuba walusebenzisa ngesikhathi ulindele ukubonwa, esikubiza nge-“Thuluzi Lokuzinqumela lwePrEP”.

6. Kungabe walisebenzisa ithuluzi lokuzinqumela? [Uma kungukuthi akaselibenzisanga ithuluzi, yeqa sonke lesisigaba]

7. Kungabe laliwusizo kanjani ithuluzi ukukusiza ukuba uthathe isinqumo mayelana nokuphuza noma ukungaphuzi i-PrEP? Sicela uchaze:

a. Kahle hle, isona siphi isahluko esisethuluzini esakusiza wathatha isinqumo?

b. Iyona miphi imininingwane ethuluzini eyayiwusizo kakhulu ukukusiza ukuthi uthathe isinqumo? Kungani?

8. Yini engashintshwa noma engenziwa ngcono ngethuluzi?

a. Okuqukethwe noma izahluko zethuluzi uqobo lwalo (Uma umbambi-qhaza enenkinga ukukhumbula lokho okwakhe ithuluzi, ungamkhumbuza ngalezi):

a. Ukuvula Izi-krini (okufaka “Izizathu Ngoba Yini”)

b. Okokukhetha: PrEP, Amakhondomu, Ukwehlisa omaqondana bami bocansi, Ukwazi noma umaqondana wami unalo yini igciwane lengculaza,Noma umaqondana wami unegciwane lengculaza, uyawaphuza amakhambi egciwane lengculaza

c. i-PrEP ibhekene nePEP

d.Ake sikhulume ngokukwi-PrEP: Iyini, Kungani kuphuzwa i-PrEP, Kungabe ilivikela kahle kanjani igciwane lengculaza, Ngiyiphuza kanjani iPrEP, Okumele ukwazi nge-PrEP, Okuyiqiniso nokuNgelona Iqiniso njll.

e. Funda Ngokwengeziwe- ukubheka ongakhetha ukukhuluma ngakho nabasebenzi basekliniki

f. Ukuhlela umndeni b. Isikhathi nalapho isetshenziswa khona [Kulabo abanqabayo uma besaqala noma uma esho ukuthi akayisebenzisanga i-PrEP]

9. Ungasicabanga isimo lapho ungafuna ukusebenzisa i-PrEP? Uma kungu-yebo, ngicela usichaze.

[Kulabo abamukele sekuhambe isikhathi kuphela]:

10. Yini eyakwenza washintsha umqondo wakho wanquma ukuqala usebenzise i-PrEP?

a. Kungabe lukhona ulwazi owalutholayo, okufaka amahlebezi/amahemuhemu, akwenza washintsha ingqondo mayelana nokusebenzisa i-PrEP? Ungachaza?

b. Abantu abasempilweni yakho benza kanjani ukuba babe nomthelela esinqumeni sakho sokushintsha umqondo wakho mayelana nokusebenzisa i-PrEP? (isib. umaqondana, umndeni, noma abangane)

c. Ucabanga ukuthi ushintsho emibonweni yakho mayelana nezinga lobungozi bakuthinta ukukhetha kwakho ukuqala usebenzise i-PrEP? Kanjani?

i. Ubungozi bokuthola igciwane lengculaza

ii. Ubungozi bokuphuza i-PrEP (imithelela engemihle, ukukhathazeka ngokwempilo ngokuhamba kwesikhathi)

d. Kungabe olunye ushintsho empilweni yakho lwenza kwaba lulanyana ukuba uphuze i-PrEP? (okwasendlini, umsebenzi, ezezimali, okunye) Kanjani?

11. Kungabe kukhona okwenzekayo ekliniki okwakwenza wanquma ukuqala ukusebenzisa iPrEP? Ngitshele kabanzi. [Kwabo bonke ababambi-qhaza]:

**Indikimba 3: Owahlangabezana Nakho Ekliniki**

12. Manje sengizothanda ukukhuluma ngokuvakasha kwakho kwasekliniki. Kwakulula noma kwakunzima kangakanani ukuza ekliniki? Yini eyayikwenza kube lula noma kube nzima?

13. Chaza ukuthi kwenzakalani uma uya ekliniki uvakasha ngokwe-PrEP.

a. Kwakulula noma kwakunzima kanjani ukuthola ezosizo ne-PrEP ekliniki? Yini eyakwenza kwaba lula noma kwabanzima?

b. Uma unokokunezelela/gcwalisela, kwakuba njani uma uye ukuyothola okokunezelela/okokugcwalisela? Wawuyaye uyephi?

14. Ungachaza ngokululekwa nokwesekwa owakuthola ngendlela yokuphuza i-PrEP?

a. Watshelwani?

b. Kwakuyizini izinto owawuzithanda ngakho?

c. Kwakuyizini izinto ongazithandanga ngakho?

d. Kungabe wabezwa beyiphendula imibuzo yakho noma okwakukukhathaza? Ungangipha isibonelo?

15. Kungabe ucabanga ukuthi waphethwa kanjani ngalesisikhathi uvakashele ekliniki?

a. Unganginika isibonelo sokwakukuhle noma sokwakukubi?

b. Kungabe ucabanga ukuthi uthole ukucaswa okuthile noma ukwahlulelwa ngabasebenzi basekliniki? Uma kunjalo, ungangipha isibonelo?

16. Kungabe wawukhululeke kanjani uxoxisana ngezihloko eziphathelene nocansi, igciwane lengculaza, i-PrEP nokuhlela umndeni nabasebenzi basekliniki owawusebenzisana nabo ngesikhathi uvakashele kwa-POWER?

a. Unganginika isibonelo sesikhathi lapho wazizwa khona ukhululekile noma ungakhululekanga ukhuluma ngalezizihloko? Yini ekwenza/engakwenza uzizwe ukhululeke kakhulu?

b. Wawukhululeke kanjani ubuza abasebenzi bocwaningo imibuzo?

**isiXhosa**

**Theme 1: Ulwazi nge PrEP *[Eyabo bathe kuqalwa bavuma nabo bathe bala]***

1. Uve njani malunga nePrEP kuqala? Wazi ntoni malunga nePrEP?
   1. Uve ngayo phi okanye ngubani othe wakuxelela nge PrEP?
   2. Ukhe wava nayiphi na into malunga ne PrEP kwimidiya? (e.g. unomathotholo, kwiintengiso, kumakhasi emidiya?)
   3. Uve ntoni nge PrEP?
      1. Ngubani onokuthi ayithathe iPrEP kwaye ngoba?
      2. Ithathwa njani?
2. Yintoni ekunike umdla nge PrEP?
   1. Yintoni ekwenze wafuna ukufunda banzi malunga ne PrEP?
3. Yintoni ekukhuthaze ukuba uze ekliniki?
   1. Ingaba ukhona umntu oye wakukhuthaza ukuba uze?

**Theme 2: Isigqibo ngePrEP *[Eyabo bathe kuqalwa bavuma / bathe bala nabo bamkele kamva]***

Ndingathanda ukukubuza malunga nesigqibo sakho sokuqala malunga nePrEP:

1. Uye wagqiba kanjani ukuba ukuthatha iPrEP ikulungele wena okanye akukulungelenga ekuqaleni?
2. Kokuphi okuhle okanye okubi othe wakuhlola xa ubusenza isigqibo?
3. Ingaba abantu abasebomini bakho baye baneyiphi impembelelo kwisigqibo sakho sokuba uyithathe okanye ukungayithathi iPrEP? (umzekelo: iqabane, ikhaya, okanye abahlobo)
4. Zeziphi iinkxalabo zempilo othe wazihlola xa ubuthatha isigqibo?
   - 1. Inkxalabo okanye ngozi yokufumana iHIV
     2. Iinkxalabo zokuba uthatha iPrEP (imiphumela, ingozi empilweni zexesha elide)
5. Ingaba iimfuneko zokuba ufumane iPrEP nokuba uyithathe rhoqo zisiphembelele njani isigqibo sakho sokuba uyisebenzise okanye ungayisebenzisi?
6. ***[Ukuba lo mbuzo awucaci, nikeza imizekelo:]*** Ukufikelela kwi PrEP, ixesha olichitha ekliniki, bucala; amandla/nomdla wokuyithatha mihla le; ukulibala; indawo yokugcina ipilisi, umthwalo wepilisi, ukuhlola iHIV
7. Ingaba ulwazi olusuka kubasebenzi base kliniki lusiphembelele njani isigqibo sakho malunga iPrEP?
   1. Bayebakuxelela ntoni abacebisi okanye abezembilo.
   2. Ukhe wathetha malunga nakuphi na ukuxhalaba kwakho malunga nePreP?

**Isixhobo sokugqiba sePrEP*:*** Makhe sixoxe ngenkqubo yethablethi /Ipad othe mhlawumbi wayisebenzisa ngethuba ubulinde idinga lakho, esiyibiza ngokuba, “PrEP decision tool”.

1. Ingaba uye wasisebenzisa isixhobo sokugquba? [**Ukuba akasisebenzisanga isixhobo, litsibe lonke eli candelo**]
2. Sibe luncedo kangakanani esi sixhobo ukukuncedeni ukuthabatha isigqibo malunga nokuyithatha okanye ukungayithathi iPrEP? Nceda uchaze
   1. Ncakasana, loluphi ucandelo phakathi kwesixhobo esithe sakunceda ukuba ugqibe?
   2. Loluphi ulwazi olusuka kwisixhobo obelulolona lusebenzisekayo ekuncedeni ugqibe? Ngoba?
3. Yintoni enokutshintshwa okanye iphuhliswe ngesi sixhobo?
   1. Isiqulatho okanye icandelo lesixhobo ngokwaso ***(Ukuba umthathi nxaxheba unengxaki yokukhumbula izakhi zesixhobo, unako ukumkhumbuza ngezi)***:
   2. 1.0-3.0 **Opening screens** (Kuquka “Reasons Why”)
   3. 4.0 **Options**: PrEP, Iikhondomu, Ukunciphisa amaqabane am kwezesondo, Ukwazi ukuba iqabane lam line HIV, Ukuba iqabane lam linayo I HIV, Uyawathatha amachiza eHIV
   4. 5.0 **PrEP ukuqubisana ne PEP**
   5. 6.0-11.1 **Masithethe malunga nemenyu yePrEP**: Iyintoni, Kutheni uthatha iPrEP, Iyikhusela kakuhle kangakanani iHIV, Ndiyithatha njani iPrEP, Kufuneka wazi ntoni malunga nePREP, Nyaniso kunye Nobuxoki, njalo-njalo.
   6. 12.1 **Funda kabanzi** – Ukuhlola kokhetho lokuthetha nabasebenzi basekliniki malunga
   7. 13.0 **Cwangciso ntsapho**
   8. Isetyenziswa nini na phi

***[Eyabale ukusebenzise kwakuqala okanye ukuba uthi akakhange ayisebenzise iPrEP]:***

1. Khawube nombono wesimo apho unokufuna ukusebenzisa iPrEP? Ukuba ewe, nceda undichazele

***[Eyabo bayamkele kamva kuphela]:***

1. Yintoni ekwenze watshintsha ingqondo yakho kwaye wagqiba ekubeni uqale ukusebenzisa iPrEP?
   1. Ukhe wafumana olunye ulwazi, kuquka namarhe, akwenze ukuba utshintshe ingqondo yakho malunga nokusebenzisa iPrEP? Ungakhe uyicacise?
   2. Ingaba abantu abasebomini bakho baye baneziphi iimpembelelo kwisigqibo sakho sokutshintsha ingqondo ngokusebenzisa iPrEP? (umzekelo: iqabane, ikhaya, okanye abahlobo)
   3. Uncinga ukuba utshintsho kwizimvo zakho malunga nezinga lobungozi lube nempemelelo kukhetho lwakho lokuqala ukusebenzisa iPrEP? Njani?
      1. Ingosi yokufumana iHIV
      2. Iingozi ezisukela ekusebenziseni iPrEP(imiphumela, ingozi empilweni zexesha elide)
   4. Ingaba kukho utshintsho olulolunye ebomini bakho olwenze kwabalula ukuthatha iPrEP? (Ekhayeni, umsebenzi, ezezimali, ezinye) Njani?
2. Ingaba ikhona into eyenzeke ekliniki ekwenze ukuba ugqibe ukebeni uqale ukusebenzisa iPrEP? Ndixelele kabanizi?

**Eyabathatha inxaxheba bonke**

**Theme 3: Amava aseKliniki**

1. Ngoku ndingathanda ukuthetha malunga nokutyelela kwakho ekliniki. Bekulula okanye kunzima kangakanani ukuza ekliniki? Yintoni eyenze kwabalula okanye nzima?
2. Chaza ukuba kwenzekani xa siya ekliniki utyelela ngePrEP.
3. Kwakulula okanye kunzima kangakanani ukufumana iinkonzo kunye nePrEP ekliniki? Yintoni eyenze kwabalula okanye kwanzima?
4. Ukuba uye wafumna impinda, bekunjani xa uyokuyifumana kwakhona? Uye waya phi?
5. Ungachaza ngokhansilisho kwakunye nenkxaso oyifumeneyo malunga nokuba ithathwa njani iPrEP?
   1. Uye waxelelwa ntoni?
   2. Zintoni izinto oye wazithanda malunga noko?
   3. Zintoni izinto oye awazithanda malunga noko?
   4. Uva ngathi bayiphendule imibuze kunye neenkxalabo zakho? Ungandinika umzekelo?
6. Ucinga ukuba ubuphetheke kanjani ngethuba utyelele ekliniki?
   1. Ungandinika umzekelo okuba kokuphi okwakulungile okanye kukubi?
   2. Uva ungathi uye wafumana naluphi na ucalucalulo okanye ukugwetya ngabasebenzi basekliniki? Ukuba kunjalo, ungandinika umzekelo?
7. Ubukhululeke kangakanani na ekuxoxeni malunga nezihloko zesondo, HIV, PrEP, kunye nocwangciso ntsapho nabasebenzi basekliniki osebenzisana nabo ngethuba lokutyelela kwakho uPOWER?
   1. Ungakhe unikeze ngomzekelo wexesha apho ubuzive ukhululekile okanye ungakhululekanga ukuthetha ngezi zihloko? Yintoni ekwenza okanye enokukwenza uzive ulula?
   2. Ubukhululeke kangakanani ubuza abasebenzi besifundo imibuzo?

**Theme 4: Amava okusebenzisa iPrEP *[Eyakhe nawuphina othe wasebenzisa i PrEP: abayisebenzise kuqalwa, abayisebenzise mva, abaqhubekekayo, nabangaqhubekekiyo kwakunye nabathe baba netsholongwane beyisebenzisa]***

Ngoku ndingathanda ukuthetha malunga namava akho okusebenzisa iPrEP.

1. Bekunjani ukuthatha iPrEP kuwe?
   1. Ndixelele malunga nethuba lokugqibela uthatha ipilisi…kwakunini, kuxesha liphi, wawusenzani, kwaye wawundawo ni?
   2. Ubuzigcina phi iipilisi zakho? Ikhona imiceli mngeni ngokuzigcina?
2. Ingaba ukuthatha iPrEP kukwenze waziva njani ngesiqu sakho?
   1. Uzive njani ngokwasenyameni ? (Ikhona imiphumela? Ikhona imiphumela emihle)
   2. Uzive njani emoyeni?
3. Uyithatha kangakanani iPrEP?
4. Zeziphi izinto ezithe zabaluncedo/ezenze kwabalula kuwe ukuthabatha iPrEP? Nceda uchaze nawaphi na amaqhinga othe wawasebenzisa ukunceda ukuthatha iPrEP.
5. Zeziphi izinto ezithe zenza kwabanzima kuwe ukuthatha iPrEP?
   - 1. Uye wamelana njani nale miceli-mngeni?
6. Ucinga ukuba ukhuseleke kakuhle kangakanani nge # lomthamo othe wawuthatha?
7. Ndixelele ngexesha apho uye waphosa ukuthatha ipilisi yePrEP.
8. Yintoni ekwenze ukuba uphose?
9. Uye wathetha nabasebenzi base kliniki malunga nayo? Ukuba hayi, kutheni? Ukuba ewe, baye bathini? Iye yaba luncedo?
10. Yintoni enokunceda ukuba ikhusele ukuphosa uthatyathoo lwepilisi kwakhona?

***[Eyabo bangaqhubekiyo okanye abaqhubekayo ababonisa izikhewu ekuyisebenziseni]***

1. Ukuba uthe wayeka ukusebenzisa iPrEP nokuba lixesha elingakanani na, okanye akuyisebenzisanga ipilisi ubuyithathile, yintoni ekwenze wayeka?
2. Ukhe wafumana ulwazi oluthile olungelunye, kuquka amarhe, athe akwanze ukuba utshintshe ingqondo yakho malunga nokusebenzisa iPrEP? Ungacacisa?
3. Ingaba abantu abasebomini bakho bakuphembelele njani kwisigqibo sakho sokuyeka ukusebenzisa? (e.g., iqabane, ikhaya, okanye abahlobo)
4. Ingaba lukhona utshintsho obelukhona ebomini bakho (ekhayeni lakho, emsebenzini, kumvuzo, ezinye) eziye zenza kwalukhuni ukuthatha iPrEP?
5. Ucinga ukuba utshintsho kwizimvo zakho malunga nezinga lobungozi bube nempembelelo kukhetho lwakho lokuyeka ukusebenzisa iPrEP? Njani?
   - 1. Ingozi yokufumana iHIV
     2. Iingozi zokuthatbatha iPrEP (imiphumela, ingozi empilweni zexesha elide)
6. Ingaba ukuthatha ipilisi mihla le bekunzima? Nceda uchaze ngobunzima bakho.
7. Ingaba zikhona ezinye izizathu ezibangele ukuba uyeke ukuthatha iPrEP?
8. Ingaba kukho into eyenzekileyo ekliniki ebangele ukuba ugqibe ngokuyeka ukusebenzisa iPrEP? Ndixelele kabanzi.
9. Ucinga ukuba uyakuyisebenzisa iPrEP kwixa elizayo? Ngoba?

**Theme 5:** **Ezobudlelwane**

1. Ngoku masithethe malunga nezobudlelwane bakho. Ingaba kukho ubudlelwane onabo ngoku? (Iqabane eliphambili, iqabane lethutyana)
   1. Ndixelele malunga neqabane lakho eliphambili nokuba ubudlelwane benu bunjani.
   2. Unawo amanye amaqabane?
      1. Ndixelele malunga nobudlewane bakho nawo.
   3. Kubudlelwane okubo, ingaba wakhe wathetha nge HIV neqabane okanye amaqabane akho?
2. ***[Ukuba Ewe]*** Ndixelele banzi malunga neengxoxo ebenithe nanazo.
3. Ingaba nikhe nalwenza uvavanyo lwe HIV kunye namaqabane okanye neqabane lakho?
   1. ***[Ukuba ewe]*** Uyasazi isimo sovavanyo lwe HIV seqabane okanye amaqabane akho?
   2. Ingaba ukhe waba namava obudlobongela kubudlelwano bakho namaqabane okanye neqabane lakho? (ngokomzimba, ngokwemvakalelo, ngokwesondo, ngokwengqondo)
4. Ukhe waxelela mntu uthile ukuba usebenzisa iPrEP okanye ucinga ukuyisebenzisa iPrEP? Ngoba?
5. ***(Ukuba ewe)*** Ngubani owamxelelayo? (qabane, umhlobo, usapho) Kwaye ihambe njani loo nto?
6. Ungakhe undixelele ukuba abantu abasebomini bakho basabele njani ekusebenziseni kwakho iPrEP?
7. Zezikabani iintshukumo ezibalulekileyo kuwe?
8. Ngubani owakuxhasayo ekuthatheni iPrEP? Njani?
9. Ngubani othe akakukhuthaza ekusebenziseni kwakho iPrEP? Baye bathini?
10. Ingaba kukhona obalulekileyo oyifihlayo yona (PrEP) kuye?
    - 1. Ingaba ukhona obalulekileyo ongamxelelanga? Ngoba?
11. ***[Ukuba hayi]*** Yintoni ekwenze wagqiba ekubeni ukusebenzisa kwakho iPrEP ibe yimfihlo?
12. Yeyiphi inkxaso okanye ingcebiso othe wayifumana kubasebenzi basekliniki ngokuthetha ngePrEP kwiqabane/ kumaqabane /kusapho lwakho?
13. Bakuxelela ntoni? Bakuxelela ukuba wenze njani nokuba uyithethe njani kusapho nakwiqabane/amaqabane akho?
14. Bayisingatha njani imiceli mngeni obujongene nayo?
15. Ngaziphi indlela inkxaso eyaba luncedo/yonelisa?
16. Inkxaso ingenziwa njani ukuba ibe ngcono?
17. Loluphi ulwazi owalufumanayo malunga nePrEP ukwabelana nosapho lwakho okanye iqabane lakho?
18. Zeziphi ezinye izinto ongathanda ukuba wabelane ngazo namaqabane/ qabane okanye nosapho?

***[Ukuba ukhe wayithatha iPrEP]:***

1. Ingaba ukusebenzisa iPrEP kwachaphazela nabuphi na ubudlelwano bakho nosapho? Njani?
2. Ingaba ukusebenzisa iPrEP kwachaphazela ubudlelwane bakho kwezesondo? Njani? (Umzekelo. Ukuthatha isigqibo, amandla, uthetha-thethwano)
   1. Kunjani ngokusebenzisa ikhondomu namaqabane akho ezesondo? Itshintshile loo nto?

**Theme 6: Ibala kunye nobandlululo**

1. Ngoku ndingathanda ukuba ndithetha malunga nokuba abantu ekuhlaleni bathini na nge PrEP. Ungakhe undixelele nangaziphi na izinto okhe wazivi ekuhlani kwakho malunga ne PrEP okanye olu phando? ***[Nantsi eyona ndlela achaza ngayo ukuhlala – inokuba bubumelwane, ontanga, njalo-njalo]***
   1. Zeziphi izimvo namarhe okhe wawava ejikeleza?
   2. Uzive kubani ezi zinto?
   3. Uziva njani malunga noku ukuvileyo?
   4. ***[Ukuba bubuxoki]*** Yintoni ocinga ukuba yeyona ndlela ifanelekileyo yokushukuxa ezi zimvo/lamarhe ?
      1. Ngubani okufuneka aziqwalasele?
      2. Ingaba ikhona into eyenzileyo ikliniki okanye engayenza kubahlali ukuqwalasela amarhe okanye amabala?

**[Ukuba ebesebenzisa iPrEP]:**

1. Ungakhe undixelele malunga nawo nawaphi na amava oluntu amahle namabi othe waba nawo kuba usebenxisa iPrEP.
   1. Ingaba bathini?
   2. Ngubani othethe ezo zinto? Khawundixelele banzi ngohlobo lwaba bantu. (Iqabane, usapho, abahlobo, abantu basekuhlaleni, abezempilo).
   3. Uziva njani ngezo zinto bazithethayo?
      1. Ucinga ukuba izinto abazithethayo ziyinyaniso okanye bubuxoki? Ngoba?

**Theme 7: Abosulelekebeyisebenzisa *[Eyabo kuphela]***

Ukuba kulungile, ndingathanda ukuthetha ngesimo sakho seHIV

1. Ngokwezimvo zakho zeziphi izinto ezaba negalelo ukuba ufumane iHIV?
   1. Ungakhe undixelele kabanzi ngethuba elithile okanye umcimbi ocinga kulapho owafumana khona iHIV?
   2. Ngaphambi kokuba wosuleleke, ukhe waba namava awo naluphi na utshintsho ebomini bakho, ngokuziphatha, okanye izimo ezinokuba kanti zenyusa amathuba akho okufumana iHIV?
      1. Utshintsho lokunamathela kwiPrEP
      2. Utshintsho kwezinye izithintelo zokuziphatha
      3. Utshintsho kubudlelwana
   3. Uva kanjani ngokuba ube kuPOWER kuye kwaba negalelo kubungozi kuwe bokufumana iHIV?
   4. Uva kanjani ukuba kwi PrEP kusenokuba kuye kwaphembelela amathuba akho wokufumana iHIV?
2. Yeyiphi inkxaso othe wayifumana kubasebenzi basekliniki ngexhesha ufumana iziphumo zovavanyo lweHIV?
3. Ingaba kwaba luncedo? Ukuba ewe, njani? Ukuba hayi, zingaphuculwa njani?
4. Ndixelele ngamava akho ngokufumana unakekelo lwe HIV
5. Ukhe wathunyelelwa kwiklinikhi yokunakekela iHIV?
6. Ukhe waba uyile kwikliniki yonakekelo lwe HIV?
   - 1. ***[Ukuba ewe]*** bakuxhasa njani abasebenzi bePrEP ekliniki ngokuya kwakho khona?
     2. Ingaba uyayisebenzisa ART ngoku?
     3. ***[Ukuba ewe]*** Anjani amava akho wokuqala ART***?***

**Theme 8: Ingcebiso [ Abantu abaqhubekekayo, abangaqhubekekiyo, nabo bathe babanentsholongwane beyisebenzisa bekwisifundo iinyanga ezi 3 okanye ngaphezulu]**

Ngoku ndingathanda ukufunda kabanzi malunga ngokuthethileyo okanye ozokuthetha kwabanye abafazana malunga nePrep.

1. Ingaba ukhe waba nokucebiso abanye abafazana malunga nokusebenzisa iPrEP?
   1. ***[Ukuba ewe]*** Ukhe wathetha nabani? Kweyiphi indawo? (e.g. esikolweni, kumakhasi okuncokola, kumaqela ekuhlaleni)
   2. ***[Ukuba hayi]*** Kutheni?
2. Ngcebiso zini okhe wazinika abanye abafazana malunga nePrEP? ***[Interviewer: Ukuba othatha inxaxheba akazange ache anikise ngengcebiso, mbuze ukuba abenombono wokuba angathini na xa enokuthi abuzwe.]***
3. Ayithathe kusini na?
4. Yintoni ekufanele ayazi?
5. Kumele amelane njani nemiceli mngeni anokuthi ahlangane nayo ngokusebenzisa iPrEP?
6. Ungamcebisa ukuba axelele abanye abantu (iqabane, ikhaya, abahlobo) malunga nokusebenzisa kwakhe iPrEP?
7. Loluphi utshintsho ebomini bakhe anokulilindela ukuba usebenzisa iPrEP?
8. Yeyiphi ingcebiso onokumnika yona malunga nokusebenzisa iikhondomu ngexa esebenzisa iPrEP?

**Theme 9: iPrEP kwilixa elizayo [Abantu abaqhubekekayo, abangaqhubekekiyo, nabo bathe babanentsholongwane beyisebenzisa bekwisifundo iinyanga ezi 3 okanye ngaphezulu]**

Ngoku ndifuna sithethe malunga nokuba ukuhanjiswa kwePrEP kunokuba njani na kwixesha elizayo.

1. Zeziphi iindawo okanye iingingqi ezingabalula kwaye zikhuseleke ukuya kuzo ukuyokufumana iPrep kuzo?
   1. Uzimisele ukuhamba kangakanani ukuya kufumana iPrEP?
   2. Lingaba lide kangakanani ixesha elikufaneleyo ukulindela ukubizelwa idinga lePrep ekliniki?
   3. Zeziphi indawo kwaye iiyure ezinokubalula kuwe ukufumana iPrEP?
2. Angenziwa njani amava ekliniki ukuba abengcono?
3. Zingenziwa njani iinkqubo zekliniki zibengcono?
4. Ingenziwa njani indawo okanye ingingqi yekliniki ibengcono?
5. Kufanele inkqubo yokugcwalisa kwakhona yenziwe njani ngcono?
6. Luhlobo olunjani lomnikezi ongamkhetha ukuya kuye ukuba akubhalele amayeza ePrEP kunye nokugcwalisa kwakhona?
7. Ndicela undichazele ngamava akho apho owaziva khona ukhululekile nomnikezeli ***[ukucacisa iimpawu zomnikezi akhululeke kuye]***
8. Yintoni ongayitshintsha ngabasebenzi basekliniki ukwenza amava akho akhululeke ngakumbi?
9. Banganceda njani abasebenzi basekliniki kubafazana ukugcina iPrEP ikhuselekile kwaye iyimfihlo ekhayeni?
10. Loluphi ulwazi okanye ukhanselishwa ocinga ukuba lungaluncedo kubafazana abafuna ukuthatha iPrEP?
11. Yeyiphi inkxaso yentlalo esuka kwiqabane, abahlobo, usapho, abafazana abayifunayo ukubanceda bathathe iPrEP?

***[Bonke abathatha inxaxheba]***

**Theme 10: Njengoko sizakugqiba**

1. Unazo ezinye iingcinga okanye imibuzo? ***[bhala phansi yonke imibuzo kwaye qiniseka ukuba uyayiphendula okanye ubhekisele umthathi nxaxheba njengoko kufuneka]***
2. Ingaba ikhona eminye imibuzo obufuna ukuyibuzwa ngokuphucula ukunikezelwa kwePrEP okanye usebenziso olulula?
